# Crash test-based assessment of injury risks for adults and children when colliding with personal mobility devices and service robots

**DOI:** 10.1038/s41598-022-09349-9

**Published:** 2022-03-28

**Authors:** Diego Paez-Granados, Aude Billard

**Affiliations:** Swiss Federal Institute of Technology in Lausanne, EPFL, Institutes of Microengineering and Mechanical Engineering, 1015 Lausanne, Switzerland

**Keywords:** Mechanical engineering, Scientific data, Applied physics

## Abstract

Autonomous mobility devices such as transport, cleaning, and delivery robots, hold a massive economic and social benefit. However, their deployment should not endanger bystanders, particularly vulnerable populations such as children and older adults who are inherently smaller and fragile. This study compared the risks faced by different pedestrian categories and determined risks through crash testing involving a service robot hitting an adult and a child dummy. Results of collisions at 3.1 m/s (11.1 km/h/6.9 mph) showed risks of serious head (14%), neck (20%), and chest (50%) injuries in children, and tibia fracture (33%) in adults. Furthermore, secondary impact analysis resulted in both populations at risk of severe head injuries, namely, from falling to the ground. Our data and simulations show mitigation strategies for reducing impact injury risks below 5% by either lowering the differential speed at impact below 1.5 m/s (5.4 km/h/3.3 mph) or through the usage of absorbent materials. The results presented herein may influence the design of controllers, sensing awareness, and assessment methods for robots and small vehicles standardization, as well as, policymaking and regulations for the speed, design, and usage of these devices in populated areas.

## Introduction

Autonomous mobile devices, already share the floor with pedestrians offering services like personal mobility devices (PMD) for people transport (ZMP, Japan), or mobile service robots (MSR) for goods delivery (Starship, US), cleaning in malls and airports (Bluebotics, Switzerland), and patients support in hospitals (Aethon, USA). By the end of 2021, 39,000 units worldwide will have deployed^1^, offering huge social and economic benefits. Notwithstanding, these semi-autonomous and autonomous mobile devices raise concerns regarding safety, regulation, and responsibility chain when accidents occur involving bystander pedestrians. Similar to self-driving vehicles^2^, the control systems and design of these new devices need to be regulated, certified, and evaluated before deployment based on factual risk data and methods that ensure agreed levels of risk for pedestrians.

**Figure 1:**
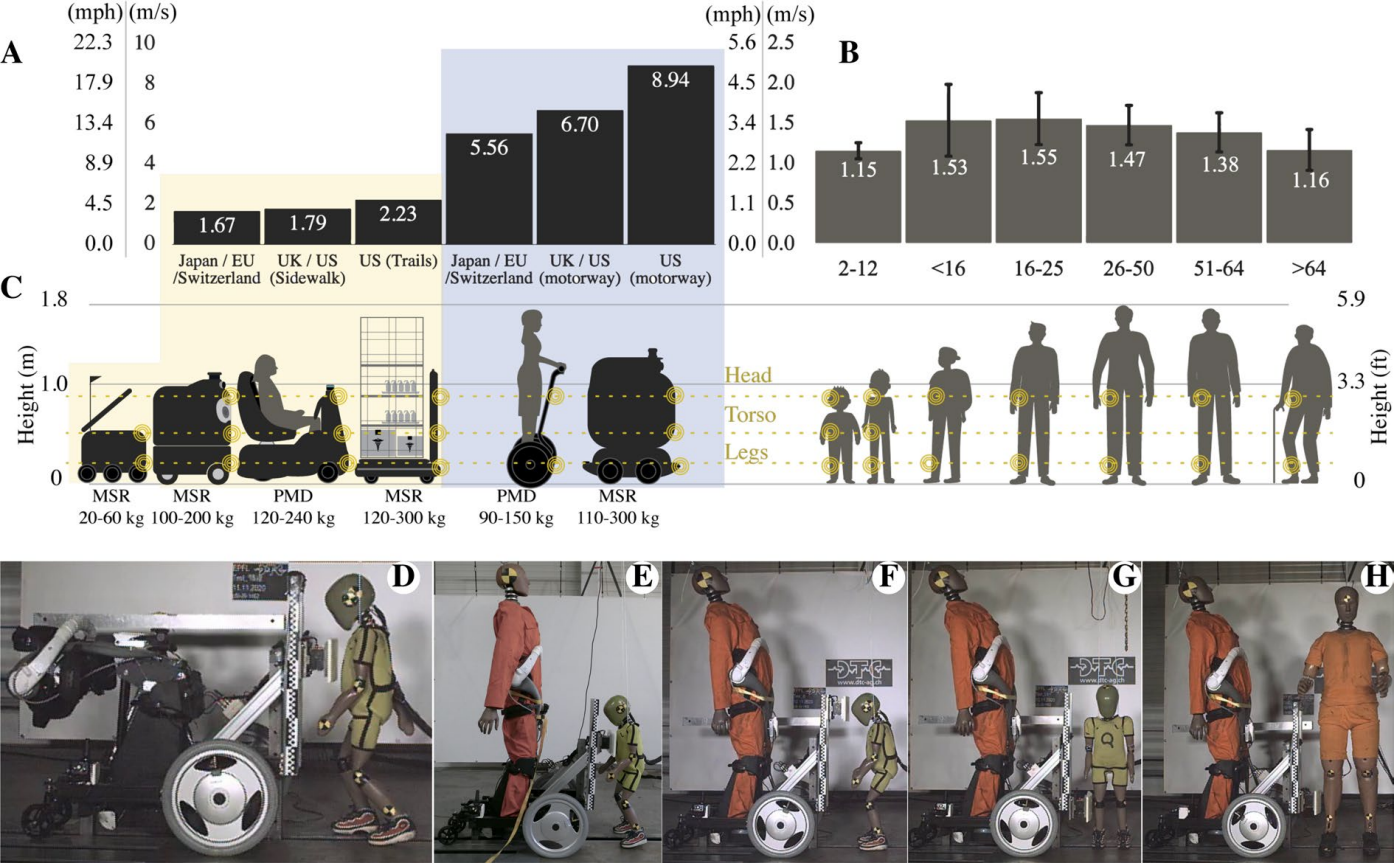
Pedestrians’ and robots average speed. (**A**) Normative speed for vehicles in pedestrian lanes and motorways in the US, Europe, and Japan (2021). (**B**) Pedestrians’ average walking speed from 2-year-old children to elderly adults. (**C**) Height comparison between pedestrians and mobile service robots (MSR) and personal mobility devices (PMD). (**D**) Child pedestrian collision at the thorax with a 60 kg MSR. Subsequent tests with a PMD 133 kg impacting at the child’s thorax (**E**), head (**F**), legs (**G**), and with an adult pedestrian at the lower legs (**H**).

A decade of research has been devoted to devising strategies for minimizing the risks of collisions of industrial robots with coworkers. The strategies range from ensuring that the robot remains always within safety zones^3,4^, to having the robot absorb most of the impact through passive or active mechanisms to control its compliance^5^. Standards such as ISO 15066:2016^6^ have been established to enforce the operational safety of these robots prior to usage. To minimize risks of severe injury, these ISO standards restrict the robot’s speed to ensure that the impact force remains below an acceptable pain threshold. Regarding safety concerns posed by road vehicles, regulations were first directed at the safety of vehicle occupants, but recent efforts in Europe^7^ and Japan^8^ include pedestrian safety in the assessment. US^9^ and European^10^ regulations are all based on risks estimated when the vehicle drives above 8.33 m/s (30 km/h, / 18.6 mph), a speed much higher than that of small service vehicles. The closest regulation for MSRs/PMDs might be ISO-22737:2021^2^, a standard of functional safety assessment protocols for autonomous vehicle control systems driving under 8.89 m/s (32 km/h/19.8 mph). However, this standard defines all pedestrians as vulnerable road users, effectively focusing on road vehicles rather than autonomous vehicles on pedestrian lanes.

PMD accidents have increased significantly in recent years. In the US alone, 14.651 e-scooter accidents were reported in 2018. This is 1.82 times more than the previous year^11^. Moreover, usage of motorized PMDs has triple risks of severe injury compared to usage of other human-powered devices^12^, and double accident numbers, as is the case in Singapore^13^. Notwithstanding, commercially available PMDs have been inconsistently regulated across countries (Fig. 1A). Some new regulations on the use of rideable PMDs have published speed limits up to 6.9 m/s (25 km/h/15.5 mph) on pathways in Singapore^14^, and Australia^15^, whereas some US states, such as New York^16^, have banned them from sidewalks. In contrast, delivery robots have been considered safe to operate in pedestrian areas throughout many US states, such as Pennsylvania and Washington. These regulations are meant to protect both the mobility device’s user and operative environment^17^. However, estimating the true risks posed specifically to bystanders is difficult for lack of or limited data in this regard. Bystanders may be particularly vulnerable owing to their physical fragility compared to sturdy materials making up MSR/PMD^18^. Children may be particularly at risk due to their small weight and height as compared to the mobility device. Current data and evaluation methods for designing industrial robots^6^ or vehicles^2^ are poorly suited for evaluating the risks generated by small mobile devices, which differ in the targeted operational environment and fall outside the range of speed and mass of typical industrial robots and larger road vehicles. Formal safety methods, standards, and regulations for MSR and PMD have been lacking^19^. In order to set appropriate limits to operational velocities and design specifications for MSR- and PMD-type devices operating in pedestrian areas, realistic injury risks needs to be evaluated. However, today, data from crash tests assessing the safety of robots were predominantly done with industrial robot arms^3^ and focused on upper-body injuries in adult operators.

This study estimates risks of injury faced by adults as compared to children in blunt impact with an MSR and a PMD. We investigated the influence of the operational speed and mass of the mobility device on injury risk and analyzed which body parts are most at risk of injury for these different categories of pedestrians (Fig. 1). We contrast blunt impact when the vehicle hits an adult pedestrian dummy to when it hits a child dummy (see Fig. 1). We further compare injury risks when the vehicle travels at low, moderate and high speed, within the range of current speed limits (6–8 km/h/3.7–5 mph). Additionally, we estimated the risk of secondary injury following a fall post-impact, to which the elderly^20,21^ and children are particularly vulnerable^22^. We provide all data and computational methods on an open-access dataset^23^.

Further, in an effort to provide guidance on the design of MSR/PMD to minimize risks, we evaluated how injury risks vary depending on the choice of construction materials, mass and operational velocity. While no combination of these parameters can lead to risk zero, risks can be reduced to less than 5% for speeds lower than 1.41 m/s (5.0 km/h/3.1 mph) and when using highly absorbent material for coverage.

We found that blunt impacts with MSR/PMD traveling at both moderate and high speed, carry a high risk for serious injury in pedestrians both at blunt impact and post-impact. Sensitive body parts, namely the head and chest, are particularly at risk in children as they come in direct contact with the device. Mitigation strategies to reduce risks would require guidelines on design and construction choices and call for uniform regulations on MSR/PMD’ operating speed when navigating in pedestrian areas.

## Results

Experiments were conducted at the Dynamic Test Center AG, in Biel, Switzerland, using Qolo—a mobility device for individuals with lower body disability^24^. Two experimental settings were contrasted. The first setting involved the use of the mobility device in stand-alone mode with a weight of 60 kg, similar to commercially available delivery and cleaning MSRs. The second setting involved the use of the robot as a PMD carrying an adult dummy passenger with a total weight of 133 kg, similar to e-scooters and other wheelchairs (Fig. 1C), see Supplementary Table  S1.

The robot travelled at a constant speed and collided frontally or sideways with either a child dummy (Fig. 1D–G), akin to a 3–5-year-old child (1.0 m, 15 kg/$3' 2''$, 33 lb), or a male adult dummy (Fig. 1H), representative of the 50th percentile of the US population in both height and weight (1.8 m, 81.5 kg/$5' 10''$, 179 lb). The impacting velocities were recorded at 3.1 m/s (11.1 km/h/6.9 mph), 1.5 m/s (5.4 km/h/3.3 mph), and 1.0 m/s (3.6 km/h/2.2 mph). Low velocities correspond to a vehicle travelling at low speed hitting a static pedestrian. A high speed of 3.1 m/s corresponds to a situation in which both the vehicle and the pedestrian move, e.g. a walking pedestrian, with a vehicle travelling close to the speed limit (see “Methods” section).

### Risk of head injuries in child pedestrians

**Figure 2:**
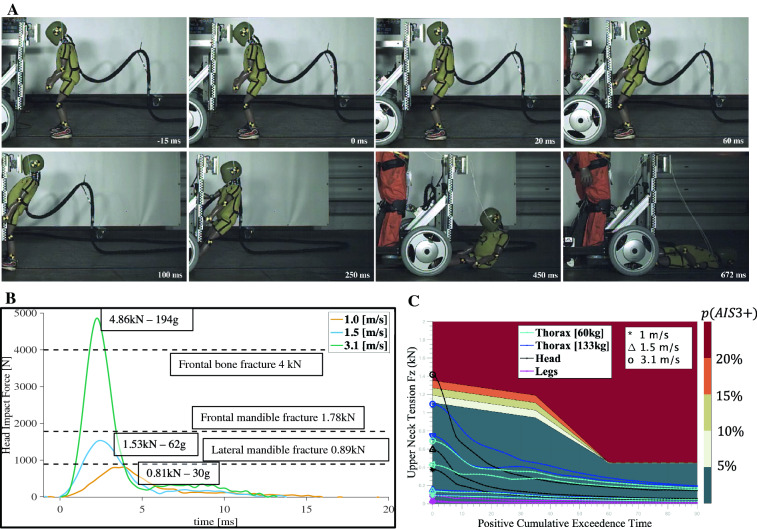
Child pedestrian injuries from a head impact. (**A**) Time-lapse of a mobile service robot (133 kg) impacting the head of a child dummy (2.7 kg). (**B**) Measured head-to-robot resultant force when varying the velocity at impact compared to reference results for bone fracture values from adult head impacts^53^. (**C**) Injury estimation at the neck of the child dummy based on exceedance of neck extension force under tension measured on the scale AIS3+ at all impact locations.

Risks following impacts to the head (Fig. 2) were assessed using the head injury criteria (HIC15), a metric of cumulative exposure to accelerations within 15 ms, and used to estimate the associated probability of a serious injury, that is, injury of level 3 in the abbreviated injury scale (AIS)^25^. Collisions at 1.0 m/s (3.6 km/h/2.2 mph) and 1.5 m/s (5.4 km/h/3.3 mph) resulted in very low HIC (HIC15 values of 10 and 44, respectively). Whereas collisions at 3.1 m/s (11.1 km/h/6.9 mph) resulted in an HIC15 of 549 (Fig. 2B) which corresponds to a 14% probability of a serious injury (AIS3+). This value exceeds the 5% probability cut-off (HIC15 of 500), deemed acceptable in the automotive industry^26^ and child head injury in playgrounds^22^.

We further analyzed the risks of injuries to the neck, by computing the cumulative tension forces under extension bending moments (Fig. 2C). There again, impacts at 3.1 m/s (11.1 km/h/6.9 mph) led to a risk of serious neck injury, with measured reaching up to 1.44 kN of upper neck tension during extension. Time series data can be found as Supplementary Fig.  S3.

### Risk of thoracic injuries in child pedestrians

**Figure 3:**
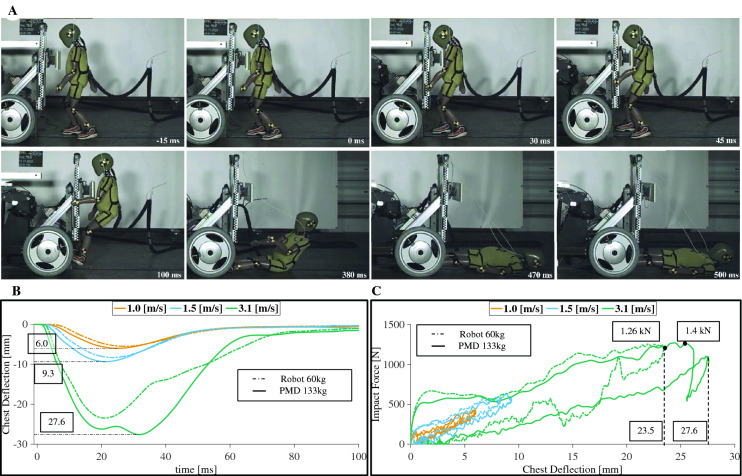
Child pedestrian injuries from a thoracic-level impact. (**A**) Time-lapse of a mobile service robot (mass of 60 kg) colliding with a 15-kg child dummy with the impact set at the chest (0.57 m from the floor). (**B**) Internal chest deformation as measured within the dummy, with a maximum deformation of 27.6 mm at 3.1 m/s (11.1 km/h/6.9 mph), equivalent to a 50% risk of serious injury. (**C**) Impact force-to-deformation ratio during the first 100 ms of the collision, showing a corridor of deformation proceeding the impact.

Impacts from a blunt unconstrained collision at the thorax at 3.1 m/s (Fig. 3A) led to chest deflections of 27.6 and 23.4 mm (Fig. 3B), and resultant peak impact forces of 1.27 and 1.26 kN, (Fig. 3C). These values correspond to the conditions of PMD (133 kg) and MSR (60 kg), respectively. These resulted in an estimated 50.8% and 32.4% probability of severe injuries (AIS3+), resp. No significant risk of serious injury was found at low (1.0 m/s) and moderate speed (1.5 m/s), see Supplementary Table  S2 and Fig.  S4.

### Risks of lower leg injuries for adult pedestrians

**Figure 4:**
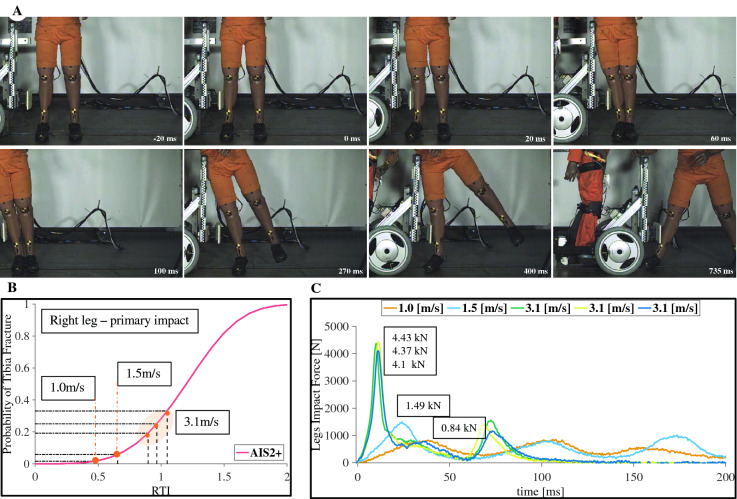
Impact with an adult pedestrian. (**A**) Time lapse of an adult collision at 3.1 m/s (11.1 km/h or 6.9 mph). (**B**) Probability of tibia shaft fracture at the measured speeds. (**C**) Force profile of the impactor measured from the robot side.

Risks of lower leg injuries (Fig. 4) were assessed using the tibia compression axial forces criteria (TCFC) and the revised tibia index (RTI), metrics, and were mapped to a probability of tibia fracture following^27,28^. Risks of moderate injury (level 2 on AIS scale) were found for all speed conditions, with a probability of 7.7%, 14.9%, and 33.2% at 1.0, 1.5 and 3.1 m/s, respectively (see Fig. 4B), the entire list of metrics can be found as Supplementary Table  S3. These results do not account for the fact that risks of fracture vary with age and gender^28^. Accordingly, women under 40 years old would have a 1.68 times higher risk for tibial fractures compared to their male counterparts. Moreover, men and women pedestrians over 70 years old would have 1.68- and 1.89-times higher risk for fractures, respectively. Results for child dummy impacts can be found on Supplementary results (see, Supplementary Fig.  S5).

### Injuries from impact to the ground, post-collision

**Figure 5:**
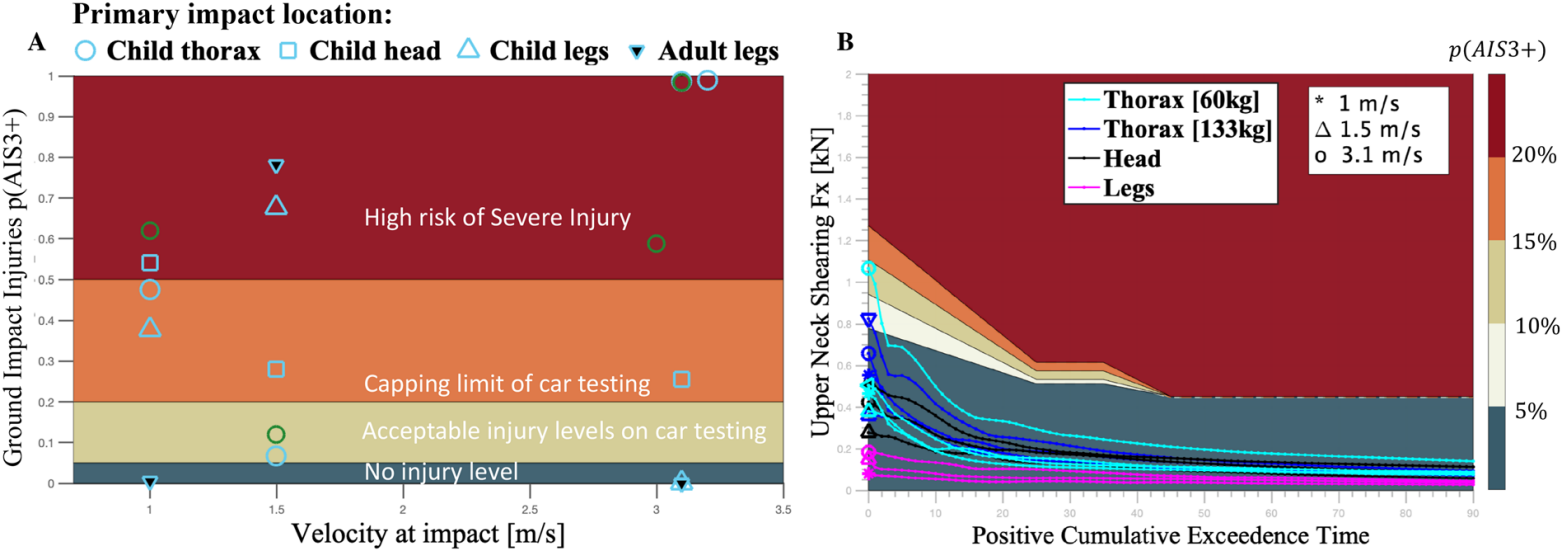
Injury risk probabilities on a fall. (**A**) Head injury criteria measured upon contact with the ground (concrete) as a secondary injury caused by the blunt impact with a robot or personal mobility vehicle. (**B**) Neck shearing forces showed an over 5% risk of serious injury for impacts on the thoracic region.

We further analyzed worst-case scenarios, namely when the collision would cause the pedestrian to fall, and computed the probability that such a fall may induce a serious injury (AIS3+). Only one out of three trials in which the adult fell would have caused a serious head injury [p(AIS3+) $> 50\%$], whereas this appears in 7 out of 14 trials in the child. Considering all trials and conditions, we find that impact on the ground, subsequent to a fall, may cause serious head injuries [p(AIS3+)] in 70% and 66% of the cases at low and high speeds, respectively, for both child and adult. Moderately severe injuries (AIS2) would occur in 20% of the cases at low speed (see Fig. 5A). However, regardless of speed, in lucky cases when the body would fold and the shoulders would hit the ground first, the fall would not lead to a head injury (HIC < 500), see Supplementary Fig.  S11. This happened in 18% of the cases. At high speed (3.1 m/s), results show a 5%, resp. 10% chance, of incurring serious neck injuries after ground impact (Fig. 55B) when the child pedestrian hits the MSR (60 kg), resp. PMD (130-kg), resp. Additional metrics can be found in Supplementary Table  S4 and Supplementary Fig.  S12.

### Design evaluation through impact and operational conditions

**Figure 6:**
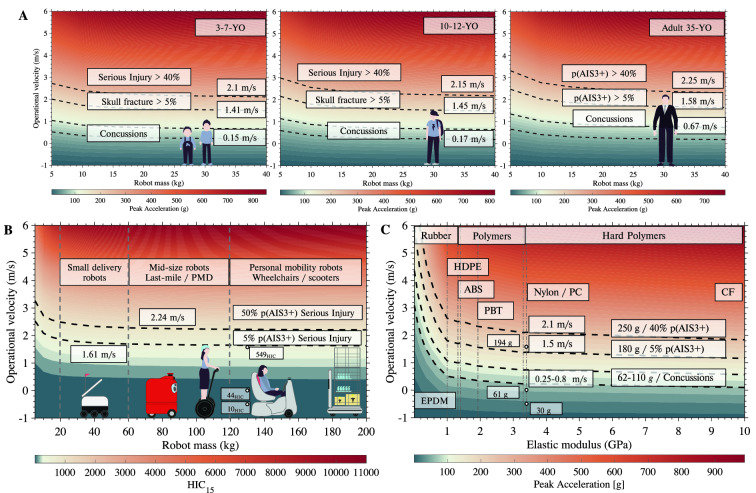
Analysis of design and operational conditions for mobile robots. (**A**) Evaluating the robot design parameters according to possible injury levels in cases of head impact comparing three groups of pedestrians. The reference values of operational velocities account for a pedestrian at 1.5 m/s (5.4 km/h/3.3 mph). Here concussions and serious injuries at 5% and 40% levels are marked by black lines. (**B**) Operational mass against velocity shows a saturation of the HIC metric over 20 kg for child head impact, reaching 90% of the maximum values at 200 kg. Measured impact points with the Q3 dummy at marked at 133 kg. (**C**) Simulating impacts of a 120 kg robot while changing material properties of the contact surface. The measured points with the Q3 dummy are marked at 3.4 GPa (Nylon).

To investigate the effects of the construction material, the robot’s mass and operational velocity on risks of head injuries, we adapted the Hunt-Crossley (HC) model^29^, as detailed in “Methods” section (see Supplementary Fig.  S1). We also simulated the role that the weight of the pedestrian would have on injury risks, so as to account for a wider population than represented by the two dummies used in our crash tests. We find that low-weight pedestrians, such as children in the age groups 3–7- and 10–12-years-old, are particularly at risk of incurring serious injuries in all conditions (Fig. 6A). For children under 18 kg (3–7 years old), if the robot impacts the child’s head, this would result in a concussion even when the vehicle moves as slowly as 0.15 m/s (0.54 km/h/0.33 mph). The 5% cut-off probability of skull fracture in children 3–12 years old is reached at a relative speed of 1.41 m/s (5.0 km/h/3.1 mph), e.g., when a walking child would hit a robot moving at a low speed of 1m/s. Such head injuries were prevalent in both low-mass (20–60 kg) and heavier (120–200 kg) MSR/PMD (Fig. 6A,B). Above 20 kg, the robot’s mass has little bearing on impact and risks of injuries remain similarly high no matter how heavy the robot. Risks reduce importantly for robot’s masses less than 10 kg.

Comparing common shell/bumper plastics used by vehicle manufacturers (see Fig. 6C), we find a 15% increased injury level at a stiffness of 10 GPa (carbon fiber) compared to polymers of 3 GPa (Nylon). In contrast, more compliant polymers (1 GPa) like HDPE reduced injuries by 29% and rubber materials (6.7 MPa) like EPDM reduced the injury risk by up to 82% for peak accelerations and 93% in the HIC index (see Supplementary Fig.  S2). Although validated with a single material in our study, the model results match those documented in^22,30^, and show up to two times decreased peak linear head acceleration when changing from 3.3 GPa (wood or Nylon) to rubber-like materials under 6.7 MPa (EPDM, playground foam).

## Discussion

The present study addressed whether blunt collisions with small size vehicles or service robots could induce injuries to a pedestrian, including children as a vulnerable population. **Child pedestrian injuries** to the head and thorax presented the most significant risk from an impact speed of 3.1 m/s (11.1 km/h or 6.9 mph), yielding **possible skull fractures, neck injuries, and chest injury**. Moreover, the probability of serious injury risk p(AIS3+) was between 5 and 50%, significantly higher than any previous study on robotics^3,31^. In contrast, impact speeds of 1.5 m/s (5.4 km/h/3.3 mph) and 1.0 m/s (3.6 km/h/2.2 mph) showed no sign of any of the previous injuries even at sensitive zones over the head and thorax of the child. Internal injuries on the head could not be assessed and would deserve further consideration. Such impacts could lead to severe brain injuries in the presence of angular acceleration^32^.

Although the data revealed 50% probability of serious injuries risks to the chest, the inference is limited, as valid injury data remains scarce for the younger populations where the only AIS3-level reported injuries are rib fractures. Blunt impact on the chest may also lead to injuries to the internal thoracic organs. Such injuries may arise even in the absence of rib fractures^25,33^ and are rated at level 4 and more on the AIS scale. Currently, insufficient data is available to estimate the risk of injuries to such internal organs from chest deformation data only. Ongoing efforts are directed at developing and validating biomechanical models that account for the physiology of children and that are specific to impacts with low-velocity vehicles, such as those considered here^22,34–36^, and allow their combination with specific body part models that better determine the effects of blunt forces.

Our results indicate that the **adult population** would most likely experience collisions at the lower legs resulting in moderate injuries, with a 33% probability for tibial fractures at the highest tested speed of 3.1 m/s (11.1 km/h/6.9 mph). Risks faced by adults as compared to children are hence much less. However, the adult population is diverse with some individuals more vulnerable than others. One limitation of the current study was our usage of a single child and adult pedestrian due to the lack of available models in other weight ranges. In particular, the elderly, women and lighter-weight people, who we found to have a 1.68 times higher risk of fractures given their bone composition^28^, were underrepresented. A comprehensive analysis of risks entailed by adults requires careful analysis and more research. Special attention should also be given to the shape and height of the vehicle in regards to risks posed to certain groups of pedestrians. For instance, pregnant women, whose belly would be at height level to many robots (see Fig. 1), may be more exposed in collision with tall vehicles.

Our collision model shows that rubber polymers (EPDM) would provide a better surface stiffness mitigating impacts even at speeds of 3.0 m/s (10.8 km/h/6.7 mph) (see, Fig. 6C). The opposite is equally valid. Higher stiffness in the impact surface could result in the same level of injury for lower speeds e.g., aluminum, or ABS covers. Data such as those may support manufacturing choices in a similar vein as what is in use for cars’ bumpers and hoods^7,8^. Although this model matches well the decreased head injury risk by material choice as reported in^22,30^, the simulation results should be used with caution, accounting the overestimated peak acceleration of 5% for the highest speed, and underestimated HIC with up to − 6% (see Supplementary Figure  S1).

Notwithstanding, our analysis shows that risks can be reduced by more than half when using more flexible material. Adopting standards, that request the usage of absorbent material for the robot’s cover in areas most at risk to get in contact with children’ sensitive body parts, would minimize risks, and be akin to existing standards for safe playground materials^22^.

**Secondary ground impact** was another injury risk found in all tests following a collision. Both the adult and child face risks of head injuries irrespective of the speed at collision. The current data provided quantified injury levels in case of head impact upon falling as a consequence of the first impact, which agree with medical records regarding falls showing that the most serious injuries result from head impacts^37^. Moreover, medical data have shown that adult populations over 65 years old are at higher risk for falls and longer recovery time^20^. Our data for child pedestrians under 15 kg similarly place them at high risk for injury from falls, which is consistent with the higher risk of falls in playground areas among < 3–4-year-old children^22^.

Although these data provided an injury probability during a fall, estimating whether an impact would cause a fall remains difficult given that neither the dummy nor simulated tests capture the dynamics and variability of pedestrians’ responses. A young and healthy pedestrian may be able to recover balance, whereas an older pedestrian with limited mobility may not. Awareness of the upcoming impact would also play a decisive role. Unexpected impacts arising from limited visibility would be more difficult to tackle. Situation-aware control approaches that modulate the robot’s speed in dense environments and when approaching vulnerable pedestrians may, hence, reduce risks.

**Impact modelling analysis** suggested that speed limit around vulnerable populations was the most efficient approach for mitigation (see, Fig. 6A), followed by hull material choice (see, Fig. 6C), where a significant decrease in the risk of injuries could be achieved.

Wider efforts are required to collect more comprehensive data on collision, so as to determine realistically safety limits. This is needed to create an ethical and legal framework that balances the economical and societal benefits while mitigating the potential risks of deploying service robots in pedestrian areas and to elaborate recommendations for their usage.

We expect these results to be used in reducing the risk on accidents. One direct approach to address this issue could be through speed limits in proximity to vulnerable populations, and regulations on surface materials tested for impact absorption (HIC < 500 and $a_{pk} < 62$ g), similar to playground areas^22^ or vehicle hoods (HIC < 1000)^7^. Moreover, similar to ISO22737-2021 for road vehicles, small-class mobility devices should be regulated on environmental awareness for maximum pedestrian speed recognition and test safety manoeuvres that minimize injury risk for the most vulnerable populations [e.g., $<5\%$ of p(AIS3+)]. This implies that automated driving systems should be able to recognize vulnerable populations through labelling of individuals according to age or gender following data-privacy guidelines in order to properly weight the validated younger children and elderly adults at risk in the control systems.

Our data found that children tended to be vulnerable to direct and post-collision impacts, with risks of severe and critical head injuries occurring in over 50% of the cases. Adults could similarly sustain severe injuries as shown in 1/3 of the cases sustaining a head impact. This is compounded by the fact that pedestrian fall injuries already impose a significant economic burden on the health care system, especially in pedestrians over 65 years old, as is the case in the US^21^. Regulations and city planning should account for these factors to balance the benefit of PMDs and service robots in pedestrian areas against the potential risks outlined.

In our simulations, we developed a method for safety assessment against head injuries using a computational model. Nonetheless, the complexity of the multi-limb human system and internal injury warrants future research on methods for embedding safety awareness into autonomous systems. Both robot design and controllers need to anticipate and account for the risk of collision with vulnerable populations beyond obstacle avoidance strategies, herewith guaranteeing regulated levels of injury during worst-case scenarios. This set of data and their analysis may also guide the design of robot controllers to mitigate risks of impact. They may also influence various fields, including traffic management, decision-making, control policies for autonomous driving, legislation, and standardization of robots.

## Methods

### Experimental design

We performed the experiments at the Dynamic Test Center located in Biel^38^, one of Switzerland’s official car crash testing facilities. The facility incorporated a controlled rail system that allowed driving the robot at the desired speed on a concrete ground. In the MSR condition, the robot was driven without a passenger. Its weight is 60 kg (132 lb). In the PMD condition, we installed an adult dummy (73 kg/lb) on board the robot Qolo and secured it similarly to what would be done for a regular passenger^24^. The robot and its dummy passenger led to a total weight of 133 kg (293 lb).

To account for the most vulnerable child populations walking autonomously, we selected the crash test child dummy (**Q3**). It is 1.05 m ($3'5''$) tall and weighs 15 kg (33 lb)^39^. This corresponds to a 3-to-5-years old child.

We used a Hybrid III dummy (**H3**) for the adult, with a mass of 81.5 kg (179.6 lb) and height of 1.8 m ($5'11''$), said to be representative of the 50th percentile of the male adult population^40^. We were, unfortunately, unable to secure a female dummy provided with all required sensors for comparison.

We initially focused on safety concerns of blunt collisions from delivery robots, PMDs, powered wheelchairs, and powered scooters. The typical height and shape of several existing PMDs and humans were considered to determine which body parts were most likely to sustain an impact (Fig. 1C). Accordingly, our findings showed that a bumper or hull of a PMD or MSR was most likely to impact the lower leg (tibia/fibula) of adults (heights between 0.25 m/5.9″). For child pedestrians, our findings showed that several mobile robots could reach all body parts. Therefore, we analyzed blunt impacts at most critical zones, namely the thoracic area (0.65 m/25.5″ height) and head (0.84 m/33″ height), consistent with previous studies for adult tests^41^, and matching locations of high instrumentation on vehicle crash testing dummies^26^. Notably, a similar risk was considered for the pelvis. Nevertheless, the limited instrumentation for child dummies at this location provides only acceleration data insufficient for blunt impact injury estimation.

Both the adult and child dummies were equipped with crash testing measurement units calibrated and tested according to the SAEJ211-1^42^. The measurement units encompassed: accelerometers at the head, neck, chest, and pelvis; a chest deflection unit; and a 6 axes force/torque sensor at the neck. Furthermore, the adult dummy model was equipped with force/torque sensors at the lower and upper tibia (4 axes), knees (2 axes), and femur (6 axes). Meanwhile, the robot was equipped with an impactor (0.4 m radius, flat profile) built of Nylon (MC-602) directly mounted on the force sensor (DP41-32-00/50 kN) placed on an add-on variable height frame (shown in Fig. 1D). All sensors were synchronously recorded on a main transducer unit recording at 20 kHz. Video recordings of all tests at 25 fps included a top, lateral, and onboard view of the impact. Moreover, the highest speed tests included high-speed video recording (1000 fps) calibrated and set up according to the SAE J211-2. The video material is included in supplementary video  S1.

### Experimental procedure

The tests were executed as follows: the robot was driven by the controlled railing system at a constant speed. The PMD impacted the child’s head, thorax, tibia and the adult’s tibia. The MSR was used for observing the effects of mass by impacting the child dummy’s chest, which was the most instrumented place in the dummy. A total of 14 impacts, over three locations (head, chest and lower legs) were recorded with the child dummy. 10 impacts using the robot in PMD condition (133 kg): to the head at a speed of 1.0, 1.5, and 3.1 m/s, resp., to the chest at a speed of 1.0, 1.5, 3.1 and 3.2 m/s, and to the lower legs at speed of 1.0, 1.5, and 3.1 m/s. 4 impacts to the child’s chest in the MSR condition (60 kg), at a speed of 1.0, 1.5, 3.0 and 3.1 m/s. The adult pedestrian was impacted five times on the lower legs. The test was repeated for speeds of 1.0, 1.5 and 3.1 m/s. Two additional tests at 3.1 m/s had to be repeated as the safety release mechanism on the pedestrian dummy failed the release, preventing the post-impact fall. For each test series, the robot’s force sensor was placed at the height corresponding to the impacted body part on the dummies (Fig. 1D–H). All speeds were verified by an external optical sensor and the closed-loop system of the railing system. All tests’ measurements were included in the ground impact injury analysis.

These impacting velocities were chosen as differential velocities between the agents at average walking speeds (Fig. 1B) colliding against each other, that is, PMDs and powered wheelchairs with a speed limit of 1.6 m/s (6 km/h/3.7 mph) set in most of Europe and Japan (Fig. 1A) and a pedestrian walking at a speed of 1.5 m/s (5.4 km/h/3.3 mph). Lower speeds were selected to identify inflexion points where no-injury levels could be reached. Similarly, the highest speed any purposefully built PMD or MSR could achieve for both pedestrian lanes and motorway operations (Fig. 1C) where speed limits reach up to 5.56 m/s (20 km/h/12.4 mph) in Japan and the EU and 8.9 m/s (32.2 km/h/20 mph) in the US and UK were selected.

The highest speed considered in the tests corresponds to a situation in which a pedestrian, walking at an average pace of 1.6 m/s (6 km/h or 3.7 mph), would hit a robot moving at a speed of 1.5 m/s (5.4 km/h or 3.3 mph). This is a realistic scenario of an undesirable meeting between a healthy adult pedestrian walking naturally in the street and a robot driving under the current speed limit.

For the child scenario, the highest speed considered in the tests corresponds to a scenario in which (a) the child is running at 1.5 m/s (3.6 km/h or 2.2 mph) and hits a robot moving at a very moderate speed of 1.6 m/s (6 km/h or 3.7 mph); or (b) the child is walking at a regular pace for her age, 1.1 m/s (3.9 km/h/2.4 mph) and hits a robot driving at 2 m/s (7.2 km/h or 4.4 mph), i.e. at the maximal speed limit.

Our analysis was restricted to the time window immediately after blunt and ground impacts measured manually from abrupt changes in acceleration and force data. Data from all sensors were recorded following the protocol in SAE-J211-1^42^. Namely, all sensors frequency over 20 kHz, and filter classes CFC180 for chest data, CFC600 for all force/torque data and CFC1000 to head and pelvis acceleration data. Both raw and filtered data are provided in an open-access dataset alongside this paper^23^.

### Injury metrics and evaluation

For each impact location, we related recorded data to injury risk probabilities and determined the risk severity by associating a probability for the impact to reach one of the 3-indices of the AIS scale from the US NHTSA, used to assess the severity of impact in automobile crash tests^9^. The three levels are denoted AIS1+ (low injury), AIS2+ (moderate injury) and AIS3+ (serious injury). In automotive crash-test assessments, one caps the deemed acceptable probability of reaching the highest index by an injury assessment reference value (IARV). For instance, if you test a new system and incur a 5% chance of p(AIS3), the system would be deemed acceptable and the associated device would be ranked as 5-stars. Conversely, a 20% chance of p(AIS3) would be deemed unacceptable, as 20% chance of serious injury is the cap limit in the EuroNCAP^10,26,43^.

The above percentages correspond to a probability distribution derived from historical data of censored stimulus of injury across different body parts that map injuries into a single scale^44^. The most recent review and analysis of these biomechanical indices of injury have been detailed in Ref.^45^.

The aforementioned reference values were validated using data for adults, which are usually representative of a specific category of adults, namely the 50th percentile of male adults in the US^46,47^. Data on other populations such as children are scarce. One approach to relate adult data to children is to scale the adults’ reference injury values to children. To do so, one maps adult parameters to equivalent child parameters using known physiological characteristics of body parts, considered as critical (failure stress at some muscles and bones, bone stiffness, and densities of some organs), and which have been documented by the European Enhanced Vehicle-safety Committee (EEVC) when developing the Q series of child dummies^25^.

In our analysis, injury risks were assessed through the following metrics: the 15-ms head injury criteria (HIC15), and the cumulative 3-ms acceleration ($acc_{3ms}$)—metrics of exposure to accelerations that relate to a skull fracture and brain tissue rupture. The cumulative neck tension and shearing forces (Nz, Ny) under extension bending moments, relate to damages to the Condylar joint, Alar ligaments, and spinal cord. The chest deflection (CD), relates to rib fractures. And the tibia index (TI), relates to the probability of tibia/fibula bone fracture. Detailed description, formulations, and IARV of each metric can be found on the Supplementary Methods description. Additionally, we evaluated moderate injury risk to the head for concussions and bone fractures using the IARV of peak head accelerations ($a_{pk}$), and impact peak forces ($F_{pk}$).

Our study analyzed impacts and compared them to the reference values provided by the EU CHILD project data for the Q series dummies currently used in the EuroNCAP^26^. It is important to highlight that only hard thresholds or IARV are set for child injuries, and no agreement on the probability of injury exists given the limited injury data available. Nonetheless, as a conservative measurement, we provided scaled probabilities of injury from adult data for each injury metric, as described for Q-dummies in Ref.^48^. Namely, the HIC15, peak head acceleration, neck forces, and rib deflection, derived from the method proposed in Ref.^46^ wherein all values are scaled back to the 50th percentile of adult humans using the following dummy parameters:

1 $$\begin{aligned} m_{Qn} = m_{H3} \lambda _{m}(Qn), \end{aligned}$$

where $m_{H3}$ is the resulting metric for an adult dummy H3; $m_{Qn}$ represents the index value for a Q series child dummy with *n* corresponding to the age model, $n= [1.5,3,6,10]$; and $\lambda _{m}(Qn)$ accounts for the scaling factor of dummy construction parameters from Q dummies to H3 provided in the EEVC project reported in Ref.^25^. Through this method, the index results from impacts were fitted to the probability distribution of adult injuries $pAIS = p(m_{H3})$.

As the most conservative approach to determine the probability of injury, we accounted for the over-stiff thoracic body of the Q dummies reported in the biomechanical fidelity analysis of Q3 dummies^49^ and the low speed of the current tests. Therefore, in this work we used the probabilities of injury scaled to the H3 dummy, which were shown to be conservative compared to the IARV given in^25,33^, i.e., all IARV had lower values when scaling directly from adult curves. Nonetheless, we included both IARV for each test as reference for injury referring to EuroNCAP values on the comparison for HIC and adding direct probabilities of injury from the scaled values on Supplementary Tables  S3 and  S4. Using the latest IARV from the American NCAP^9^ and European EuroNCAP^26,43^.

### Head injury model for collision simulations

To estimate the injury risks for a wider range of speed and masses than those tested in the experiments, we developed a simulation of the collision between a simplistic child’s head and robot, based on the HC impact model^29^ that gives an accurate account of impact forces between a robot arm and a human head^31^. Using the HIC15 and peak head accelerations as injury metrics, we fit the model’s parameters to our data and used these fit parameters to extrapolate the expected acceleration induced on a human head in the transient response of the collision when varying the speed, mass and material of the robot.

The blunt impact was modeled in a Hertzian reference frame (see Supplementary Fig.  S1), where child head and robot impactor are represented as 2D circular masses with radius $r_h$, $r_r$ and masses $m_h$, $m_r$ resp. Assuming homogeneous, isotropic, and frictionless bodies with elastic deformations, the dynamics of the deformations (*x*) of a mass *m* is given by:

2 $$\begin{aligned} m\ddot{x} = F(x) \end{aligned}$$

The deformation is computed as the relative displacement of the two centers: $x = x_r - x_h$, and the mass is the relative ratio of the two masses: $m = \left( \frac{1}{m_{r}} + \frac{1}{m_{h}}\right) ^{-1}$. The contact force *F*(*x*) was defined accordingly to Hertz force theory through the HC-model:

3 $$\begin{aligned} F(x) = { k_{hr} x^{n}+\lambda \dot{x}^m x^{n}} \end{aligned}$$

The term $k_{hr} x^{n}$ accounts for the nonlinear elastic force and $k_{hr}$ for the stiffness of the two materials at contact. $\lambda \dot{x}^m x^{n}$ denotes a nonlinear viscous force, with $\lambda$ an damping gain for the deformation dependent viscous force. The Hertz exponential *n* is an open parameter that must also be fit. The deformation equation is hence given by:

4 $$\begin{aligned} m\ddot{x} + \lambda x^{n} \dot{x} + k_{hr} x^{n} =0 \end{aligned}$$

which can be reduced to:

5 $$\begin{aligned} m\ddot{x} + k_{hr} x^{n} (1 + \alpha \dot{x} ) =0 \end{aligned}$$

with *n*, and $\alpha$ the two model parameters to be fit.

We assume an equivalent stiffness $k_{hr}$ estimated from the two ellipsoid masses in contact:

6 $$\begin{aligned} k_{hr} = \frac{4}{3}*\left( \frac{1-{\nu _r}^2}{E_r}+\frac{1-{\nu _h}^2}{E_h}\right) ^{-1}\left( \frac{1}{r_r} -\frac{1}{r_h}\right) ^{-\frac{1}{2}} \end{aligned}$$

where the $E_r$ and $E_h$ denote the elastic modulus of the material on the robot and head respectively, while $\nu _r$ and $\nu _h$ correspond to the Poisson ratio of each mass. The robot’s bumper material properties were $r_r=0.4 (m)$ constructed from Nylon MC-602 with $E_r = 3.3$ GPa, and $\nu _r = 0.41$. The impacted child skull was assumed to have a Young’s modulus $E_h = 4.7$ GPa, and a Poisson ratio $\nu _h = 0.26$ determined from Ref.^50^. All simulated material properties can be found in Supplementary Table  S5.

To determine the two open parameters of the HC model, we ran a regression. A least-square fitted model with all head impact data yielded the following optimal coefficients $n = 2.5$, and $\alpha =2.2$. with a root mean square errors of 3.4, 7.5, and 32.4 for impact speeds at 1.0, 1.5 and 3.1 m/s, respectively. The model provided a rather conservative prediction of the peak acceleration with slight overestimation errors of 9%, 2%, and 5%, respectively. Corresponding HIC estimation errors were 32%, 6%, and − 6.2%, respectively.

This model allows estimating the peak forces, peak acceleration, and, hence, to derive the injury index (HIC) resulting from an impact on the head. We chose to simulate this part in particular since it is the part most impacted in our study both at blunt impact and in secondary injury following a fall. It is also one of the most vulnerable body parts of the child and likewise of the adult. Similar models have been validated with adult collision data from studies on industrial robot manipulators^31,51,52^.

## Supplementary Information

Supplementary Information.

Supplementary Video 1.

## Data Availability

The datasets generated and analysed during the current study are available in the Zenodo repository, ***crash-testing-dataset***^23^.

## Code availability

Related code are available in the GitHub repository, ***crash-tests-service-robots*** https://github.com/epfl-lasa/crash-tests-service-robots.
