# Supplementary material for: Crash test-based assessment of injury risks for adults and children when colliding with personal mobility devices and service robots
Source: Sci Rep. 2022 Mar 28;12:5285. doi: 10.1038/s41598-022-09349-9 (PMC8960768; doi:10.1038/s41598-022-09349-9)
Supplement: Supplementary file 1 — Supplementary Information. [file 41598_2022_9349_MOESM1_ESM.pdf]

Supplementary Material
Crash test-based assessment of injury risks for adults and children when colliding with personal mobility devices and service robots

Diego Paez-Granados[1,*] **and Aude Billard**[1]

[1]Swiss Federal Institute of Technology in Lausanne, EPFL, Institutes of Microengineering and Mechanical Engineering, Lausanne, 1015, Switzerland
[*]dfpg@ieee.org

Summary

This PDF file includes:
Supplementary Methods: Detailed descriptions of the metrics used for assessing injury risks and detailed values for head impact simulations.
Supplementary Notes: Further data description of results for less severe injuries such as legs impacts and skull impacts at lower speeds.
Supplementary Figures S1 to S12
Supplementary Tables S1 to S5
Supplementary Movie Captions
Other Supplementary Materials for this manuscript include the following: Supplementary Movie S1 (Separate file)

Supplementary Methods:

Head Injury Criteria

Currently, the most acceptable predictor of brain injury and bone fracture (resulting from linear motions) is the maximum value of a normalized integral over the acceleration at the center of mass of the head with a set time window of 15ms - the head injury criteria (HIC15)[1]. A second indicator of injury is the maximum acceleration over a time window of 3ms (acc_{3ms}) after impact.

$$HIC_{15} = max_{\Delta t}\left\{\Delta t\left(\frac{1}{\Delta t}\int_{t_1}^{t_2}||\ddot{x_H}||_2 dt\right)^{2.5}\right\}, \tag{1}$$

$$a_{3ms} = \frac{1}{\Delta t}\int_{t_1}^{t_2+\Delta t}||\ddot{x_H}||_2 dt, \tag{2}$$

where $||\ddot{x_H}||_2$represents the norm 2 resultant head acceleration at a given time interval Δt below $15ms$. Injury reference injury values for non-blunt impacts on these two metrics were originally set to a HIC15 of 790 and 1000 for a 20% and 50 % probability of serious injury p(AIS3+)[2]. Whereas, the cumulative acceleration a_{3ms} injury reference value for the Q3 dummy is 81 and 99 g for a 20% and 50% probability of injury, respectively.

However, what would deem as a correct set of injury criteria for children is yet a research question. Recently, a few studies have focused on understanding the biomechanical constitution of children and subsequent injury risks of each body part such as head injury in young children[1,3] which showed that head injuries for peak skull stress in 3-YO models correlated to a HIC of 543. This goes in line with the most recent EuroNcap update where the reference value for 5% of p(AIS3+) and p(AIS4+) was set to a HIC15 of 500.

As the most conservative approach an estimated p(AIS3+) as a function of HIC15 was used based on regression function

from Ref.[4], as follows:

$$p\left(AIS3+\right)=\left(1+e^{\left(\alpha+\frac{\beta\lambda_m}{HIC_{15}}-\frac{\gamma HIC_{15}}{\lambda_m}\right)}\right)^{-1} \tag{3}$$

where the constants for $\alpha = 3.39, \beta = 200, \gamma = 0.00372$ were fitted to a logistic regression of injury data in Ref.[5]. The scaling factor for a child dummy Q3 was $\lambda_{HIC} = 0.71$[2].

In comparison, adult injury reference values are HIC15 of 700 and 1000 for a 5% and 20% p(AIS3+) respectively[6]. Currently, the EuroNcap[7] set capping acceptable limit to the 5% value (700) for passenger protection.

Chest Injury Criteria

Several metrics can be used to assess the probability of injury at the chest. The most notable and widely accepted viscous criteria for soft tissue criteria for car crashes has not been validated for children for lack of data. Therefore, the criteria used in this evaluation was the chest deflection $CD = ||\Delta x_C||_2$, where $||x||_2$ denotes the Euclidean norm over the chest, which is directly related to the probability of rib fractures. According to the IARV from the EEVC[2]), a deflection of 33 and 46 mm causes a 20% and 50% probability of serious injury, respectively. Nevertheless, this threshold was obtained for shoulder belt restraining systems and can thus be considered a more conservative and realistic comparison to using the airbag blunt impact values for the probability of serious thoracic injury p(AIS3+) from the National Highway Traffic Safety Administration (NHTSA) vehicle assessments[8] using the conversion factors for airbag ($\lambda_{CD} = 0.44$) provided by the EU CHILD project[2,9]

$$p\left(AIS3+\right)=\frac{1}{\left(1+e^{\left(10.5456-1.568(CD/\lambda_{CD})^{0.4612}\right)}\right)} \tag{4}$$

Neck Injury Criteria

The injury probability used to determine the AIS level for the neck was the maximum value out of three metrics: the maximum extension forces (F_z), the maximum shearing forces (F_x), and the maximum extension moments (M_y). This study used reference values from the EuroNCAP[10] and EEVC[2], where the 20% probability of AIS3+ is $N_z = 1.55kN$, or $My = 79Nm$, whereas the 50% probability of AIS3+ is $N_z = 1.705kN$, or $My = 96Nm$. Additionally, we used the cumulative exceedance forces to assess the prolonged exposure to a tension force during extension moment[10].

Tibia Injury Criteria

For the analysis of adult pedestrians, three metrics were used for comparison with known thresholds in different studies: 1) The TI index used in automotive crash test assessments [EuroNCAP[7] and NHTSA[8]] is given by: $TI = F_z/F_{zc} + M_R M_{Rc}$, with F_z the measured tibia proximal force in kN and $F_{zc} = 35.9kN$ a normalized reference value. M_R represents the resultant torque in [Nm] for the knee M_x, M_y as $M_R = ||M_x, M_y||_2$, while $M_{Rc} = 225Nm$ is the normalized reference value for the adult dummy H3. The IARV detailed in Ref.[6] is set to 5% of pAIS2+ for $TI = 0.4$ and 20% of pAIS2+ for $TI = 1.3$.

Second, the revised tibia index (RTI)[11] was used to determine the probability of incurring injuries at AIS2+ injury level (moderate injury) in adults. This index applies to lower extremity impacts, including impacts at the lower and upper tibia, and resulting torques at the knees, ankle, and foot. The associated probability o AIS2+ injury is computed as follows:

$$p\left(AIS2+\right)=1-e^{\left(\frac{\ln\left(RTI\right)-0.2728}{0.2468}\right)} \tag{5}$$

RTI is computed as the TI index but using the following reference values: $M_{Rc} = 240Nm$, and $F_{zc} = 12kN$, see[11].

Third, the probability of tibia fracture was determined using the compression axial force criteria TCFC. This last metric was recently developed as a Weibull distribution to determine lower limbs injury risk per age and gender[12], accounting for differences and variability in bone density over the life span. This complements metrics valid for 35-year-old adult male and used traditionally[8,13]].

$$p_f\left(amg\right)=1-e^{-\left(\frac{Ramg}{9.8617}\right)^{4.277}} \tag{6}$$

where the risk function *Ramg* for age, mass, and gender is defined as:

$$Ramg = \frac{F}{k_1} \frac{(k_0 A^2 - k_0 80A + k_2)}{(m + k_3)} \tag{7}$$

where constants $k_0 - k_3$ were defined for male and female in the groups below 40 and above 40-years-old independently in Ref.[12].

A limitation in this last metric from Ref.[12] is that the probability of tibia bone fracture was set from a single predictor based on the axial compression force. Thus, it can only provide partial results and serve mostly to compare different effects for more vulnerable populations, such as elderly adults and lighter people.

Head model simulation values

The model of the head impact was used for comparing different materials (see, Supplementary Table S4), at contact speeds ranging 0.5 to 7.0 m/s , and impacting masses ranging from 10 to 200 kg. Moreover, we evaluated the overall effective stiffness k_{hr} assuming constant properties in the skull and varying linearly the effective stiffness, which could result from structural arrangements in the robot (see Supplementary Fig. S2). Each variable in the model was evaluated independently using other parameters from real experimental data. In Fig. 6A, the head properties were assessed for children aged 3-4-YO, 6-7-YO, and 10-12-YO[3,14,15] with masses of 2.7, 3.4, and 3.7 kg. The head radius r_h used were 0.056, 0.078, and 0.08 m, respectively. The elastic modulus E_h were 4.7, 5.7, and 6.0 GPa respectively. The Poisson ratio ν_h were 0.26, 0.24, and 0.23 respectively. For comparison with an adult head, we simulated using a mass of 4.54 kg, Elastic modulus of 6.5 GPa, and a Poisson ratio of 0.22[16].

Supplementary Notes

Service robot categories:

A wealth of service robots and other small personal mobility devices have appeared on the market worldwide. It would be impossible to cover them all. Figure 1 compares a selected subset of existing mobile service robots (MSR) and personal mobility devices (PMD) that share the ground with pedestrians today in different regions of the world. We compare pedestrians and MSR/PMD in terms of their respective speed (and speed limits), height and weight. Figure 1A, speed limits for powered PMDs usage in pedestrian lanes in the US[17], Japan[18], the UK[19], and EU[20]. Speed limits for scooters, and PMDs in the US[17], UK[21], Japan[22], Australia[23], and the EU[20]. Figure 1B, Average walking speed of pedestrians aged 12 to 70 years old[24] and children <2 years old obtained from Ref.[25]. Figure 1C, From left to right: Delivery MSR [Starship Inc., USA[26]], autonomous PMD RakuRo [ZMP, Japan[27]], hospital good carrier MSR TUG [Aethon, USA[28]], Self balancing PMD [Segway, USA[29]], delivery MSR [JD.com, Beijing, China[30]]. See Supplementary Table S1, for a detailed description of each of these robots and other examples such as cleaner MSR-s [Bluebotics, Pittsburgh, USA[31]] and autonomous wheelchairs [Whill, Tokyo, Japan,[32]].

Risk of injuries in child pedestrians

Further analysis for less severe injuries to the head could be derived from the peak resultant accelerations, which showed low values of 30 and 62 *g* for the two lower speeds, respectively. Meanwhile, a peak acceleration of 194 *g* was noted at 3.1 m/s (11.1 km/h / 6.9 mph) (see Supplementary Fig. S3), which exceeded the known concussion threshold of 62.4 ± 29 *g* for young people[33])and the 5% skull fracture probability (180 *g*) — also a metric of p(AIS3+)[5].

Injuries to the neck as a second important metric for head impacts (**Fig.2C**) were analyzed by the cumulative tension forces under extension bending moments and compared with limits for serious injuries (pAIS3+) scaled for Q3 child dummies[9,34]). Shear forces in the neck showed no significant associated indices of injury. Time-series data is available in Supplementary Figure S3 C-D.

Lower leg injuries

For the **child dummy**, the impact force resulted from the blunt unconstrained lateral collision at the tibia (see **Supplementary Fig. S5**) . In the current standards and normative data for crash testing, child dummies lack measurement equipment at the lower body; therefore, we measured from the robot's force cell in the reaction forces and compared them with responses in adults. Results show that impact forces of 2.19 kN, 0.86 kN, and 0.33 kN, respectively, are incurred at 3.1m/s, 1.5m/s, and 1 m/s, respectively. In this case, we selected a lateral collision with a pedestrian on the double stance phase of the walking gate cycle, which would be the worst-case scenario for a direct blunt impact given that the weight is evenly distributed in both legs. There is no documented way to relate directly these values to a probability of injury in children. Nevertheless, these data can be compared to injury criteria applied to adults: for instance, impact forces $\geq$ 1.4 kN yield a 14.9% probability of moderated injury (AIS2+), such as tibial fractures. Detailed data in Supplementary Table S2.

On the adult dummy, our results showed that regardless of the speed, all impacts displayed a TI > 0.4 on the impacted leg (right). This reference injury value exceeded the 5% probability of fracture at the tibia, which was considered a moderate injury (AIS2+). The TI values were 1.16, 0.73, and 0.53 at speeds of 3.1, 1.5 and 1.0 m/s, respectively (**Fig. 4B**). All values were within the acceptable threshold for passenger vehicles (TI < 1.3) used in vehicle crash testing at a 20% probability of injury. The corresponding peak forces on the robot's impactor (**Fig. 4C**) reached 4.4 1.4, and 0.84 kN, at speeds of 3.0, 1.5, and 1.0 m/s, respectively. Other injury metrics are available in Supplementary Table S3.

A second injury risk was found on the indirectly impacted leg (left leg). In this case, only at a speed of 3.1 m/s (11.1 km/h / 6.9 mph). Two indicators showed the same risk level: TCFC of 3.89 kN and a TI value of 0.81. Detailed time-series data are available in the supplementary material for both legs (see **Supplementary Fig. S7–S9**).

Finally, we used a new metric proposed in Ref.[12]) for lower-limb injury risk, although limited to a single predictor from the internal force compression it accounts for age and gender. The probabilities of fracture from internal compression force alone were 1.9% for men and 3.2% for women under 40 at a speed of 3.1 m/s (11.1 km/h / 6.9 mph); whereas pedestrians over 70 years old would have a risk probability of 3.1% and 5.5% for men and women, respectively.

Injuries from secondary ground impact

All impact conditions caused the dummies to fall due to the sufficiently high impact energy of the robot to destabilize the standing pedestrian dummies. Detailed data is available in Supplementary Table S4.

After comparing head injury severity between child and adult pedestrians, we noted (see **Fig. 5A**) that only one out of three trials in which the adult fell would have caused a serious head injury [p(AIS3+) > 50%], whereas 7 out of 14 trials in which the child fell would have caused a serious injury [p(AIS3+) $>$ 50%]. Moreover, 4 out the 14 trails caused p(AIS3+) $>$ 20%, whereas 2 out the 14 trails caused p(AIS3+) $>$ 5%. No significant head injury probability [p(AIS3+) $<$ 5%] was observed in only 1 out of the 14 trials. Moreover, three tests resulted in a HIC $>$ 6000 (AIS4+ $>$ 98% - critical or life-threatening). These impacts occurred at the thorax where the child dummy was projected forward at the highest velocity transfer.

Overall, impacts above the COM of the dummy (chest or head impacts) showed moderate to high risk of injury, with those closest to the COM resulting in higher head velocities at ground impact. However, impacts below the COM (lower legs) and beyond 1.5 m/s (5.4 km/h / 3.3 mph) produced wrap projections impacting the robot's body continuously before the forward projection to the ground, thereby decreasing the final velocity of the body when coming into contact with the ground. Videos of all impacts are provided as supplementary materials.

Evaluation of design and operational conditions

Operational velocity differences in skull changes were found when varying the impacted pedestrian (**Fig. 6A**) from children aged 3–7 years old to an adult aged 35 years old. Accordingly, skull fracture probabilities over 5% started to appear from 1.41 m/s (5.0 km/h / 3.1 mph) among younger children and those aged 10 years old, whereas the same skull fracture probability would appear at 1.68 m/s (6.0 km/h / 3.75 mph) in an adult. Moreover, concussion injuries (peak resultant acceleration $> 62\ g$) in young children appeared at an operational velocity of 0.15 m/s (0.54 km/h / 0.33 mph) [differential speed $\Delta\dot{x} = 1.65$ m/s (5.9 km/h / 3.7 mph)], whereas those in adults (peak accelerations $> 110\ g$) appeared at 0.67 m/s (2.4 km/h / 1.5 mph) [$\Delta\dot{x} = 2.17$ m/s (7.8 km/h / 4.8 mph)].

The effect of the material's mechanical properties was evaluated by modulating the stiffness, Young's modulus of elasticity, comparing common shell/bumper plastics used by vehicle manufacturers as detailed in Supplementary Table S5.

Velocity at impact was found proportional to an increased injury rating (see **Fig. 6A–B**), the 5% risk of skull fracture was exceeded at 1.4 m/s (5.0 km/h / 3.1 mph) [$\Delta\dot{x} = 3.0$ m/s (10.8 km/h / 6.7 mph)], whereas the 40% risk of serious injury was exceeded already at 2.1 m/s (7.8 km/h / 4.8 mph) [$\Delta\dot{x} = 3.6$ m/s (12.9 km/h / 8.0 mph)]. This remains true for all robots over 20 kg that used Nylon's stiffness as the reference material. In contrast, the effect of variations in the robot's impact mass showed less effect with saturation on the exerted head accelerations at 20 kg (**Fig. 6B**), which reached 94% $\pm$ 0.14% of the peak acceleration of 200 kg impacting mass at all simulated velocities. The HIC15 index showed serious injuries p(AIS3+) from an operational speed of 1.6 m/s (5.7 km/h / 3.5 mph) for a mass of 60kg, with less than 5% $\pm$ 0.01% difference to the HIC at 200kg. Operational speeds exceeding 2.24 m/s (8.0 km/h / 5.0 mph) caused a 50% probability of serious injury based on the HIC15 (HIC15 $>$ 1000).

Supplementary Tables

Supplementary Table S1. Representative commercially available mobile service robots and personal mobility devices. Photos' sources: Starship[35], TUG[28], DeliRo[36], MiniUV[31], RakuRo[27], i2SE Segway[37], JD delivery robot[30].

Robot	Operational Speed (m/s)	Operational Speed (km/h)	Weight (kg)	Height (m)	Photo
Starship, USA	1.67	6.0	20 + load (30)	560	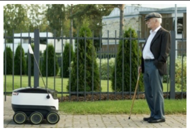
TUG - Aethon, Pittsburgh, USA	0.76	2.7	110 + load (450)	1227	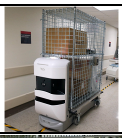
DeliRo - ZMP, Japan	1.67	6.0	110 + load (50)	108	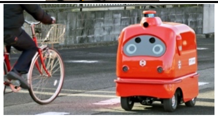
MiniUV- Blubotics, St-Sulpice, Switzerland	1.67	6.0	140	1600	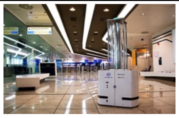
Powered wheelchair, Whill Inc. Japan	2.22	8.8	113 + person (100)	900	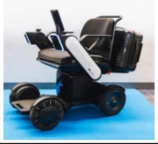
RakuRo, ZMP, Japan	1.67	6.0	120 + person (120)	1090	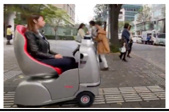
i2SE - Segway Inc., Bedford, NH, USA	5.56	20.0	48 + person (127)	900	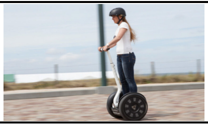
Delivery Robot, JD, Beijing, China	4.17	15.0	~120 + load (100)	1090	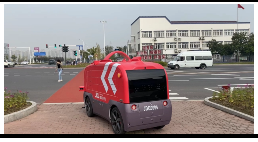

Supplementary Table S2. Impact metrics of collision for the child dummy (3-year-old / 15kg).

Impact Vel (m/s)	Mass (kg)	HIC15	Head Acc 3ms (g)	Head Peak Acc (g)	CD (mm)	Thorax Acc3ms (g)	Neck Fz (kN)	Peak Force (kN)	p(AIS3+) (%)
3.10	60.00	-	30.6	39.1	23.46	19.8	0.690	1.258	32.4
	133.00	-	28.7	39.7	27.60	21.1	1.629	1.272	50.8
		549.40	46.5	194.0			1.416	4.864	14.0
		-	30.6	42.3			0.880	2.193	0.0
3.00	60.00	-	23.3	26.0	22.70	18.3	0.577	1.261	29.3
3.20	133.00	-	32.0	50.4	25.27	19.6	1.122	1.415	40.3
1.50	60.00	-	9.5	10.1	8.31	6.6	0.766	0.599	1.1
	133.00	-	5.6	5.8	9.34	7.6	0.976	0.636	1.6
		44.20	22.4	61.6			0.950	1.534	0.1
		-	9.1	13.5			0.528	0.863	0.0
1.00	60.00	-	3.8	6.6	5.50	4.0	1.142	0.361	0.4
	133.00	-	5.4	5.8	6.00	4.6	0.525	0.439	0.5
		9.60	16.1	30.3			0.536	0.810	0.0
		-	1.5	1.7			0.792	0.331	0.0

Supplementary Table S3. Impact metrics of collision at the tibia/fibula of the adult dummy, 50-percentile male pedestrian 81.5kg.

Impact Vel (m/s)	Mass (kg)	Peak Force (kN)	TCFC right (kN)	TCFC left (kN)	TI - Right	TI - left	p(AIS2+) (%)	pF(70,M) (%)	pF(70,F) (%)
1.0	133	0.846	-0.306	-0.390	0.533	0.125	7.66	0.00	0.00
1.5		1.491	-0.703	-0.406	0.731	0.180	14.92	0.00	0.00
3.1		4.371	-0.993	-0.016	1.162	0.385	33.03	0.00	0.00
		4.435	-1.172	-3.893	1.046	0.814	33.17	1.92	6.56
		4.104	-0.853	-0.011	1.000	0.381	22.12	0.00	0.00

Supplementary Table S4. Summary of ground impact injuries with measurements at head and neck for both child and adult dummies.

Child Dummy Ground Impacts								
Impact Vel (m/s)	Mass (kg)	HIC15	Acc 3ms (g)	Peak Acc (g)	Neck Fz (kN)	Neck Fx (kN)	p(AIS3+) (%)	p(AIS4+) (%)
3.1	60	6061	32	121	0.552	1.065	98.5	98.9
	133	6297	255	599	0.753	0.659	98.7	99.1
		753	137	211	0.235	0.422	25.6	12.8
		44	45	64	0.880	0.185	0.3	0.0
3.0	60	1443	177	275	0.355	0.505	58.8	47.4
3.2	133	6668	287	570	1.052	0.826	98.9	99.3
1.5	60	513	124	163	0.766	0.380	12.0	3.8
	133	404	112	151	0.976	0.360	6.7	1.5
		795	132	228	0.950	0.278	28.0	14.7
		1718	155	360	0.528	0.149	67.7	58.8
1.0	60	1532	167	302	0.192	0.466	61.9	51.4
	133	1169	163	251	0.355	0.554	47.5	34.0
		1321	175	258	0.267	0.508	54.1	41.7
		971	144	243	0.792	0.079	37.7	23.6
Adult Dummy Ground Impacts								
1.0	133	248	84	153	-	-	0.4	0.0
1.5		3065	164	548	-	-	78.2	73.0
3.1		101	64	85	-	-	0.0	0.0

Supplementary Table S5. Material properties used on simulations of blunt impact.

Material	Young's Modulus (GPa)	Poisson ratio
EPDM	0.0067	0.50
HDPE	1.00	0.46
PP	1.33	0.42
ABS	1.40	0.29
PBT	1.93	0.39
Nylon	3.30	0.41
PC	3.40	0.34
CF	10.00	0.30
Al	368.00	0.35

Supplementary figures

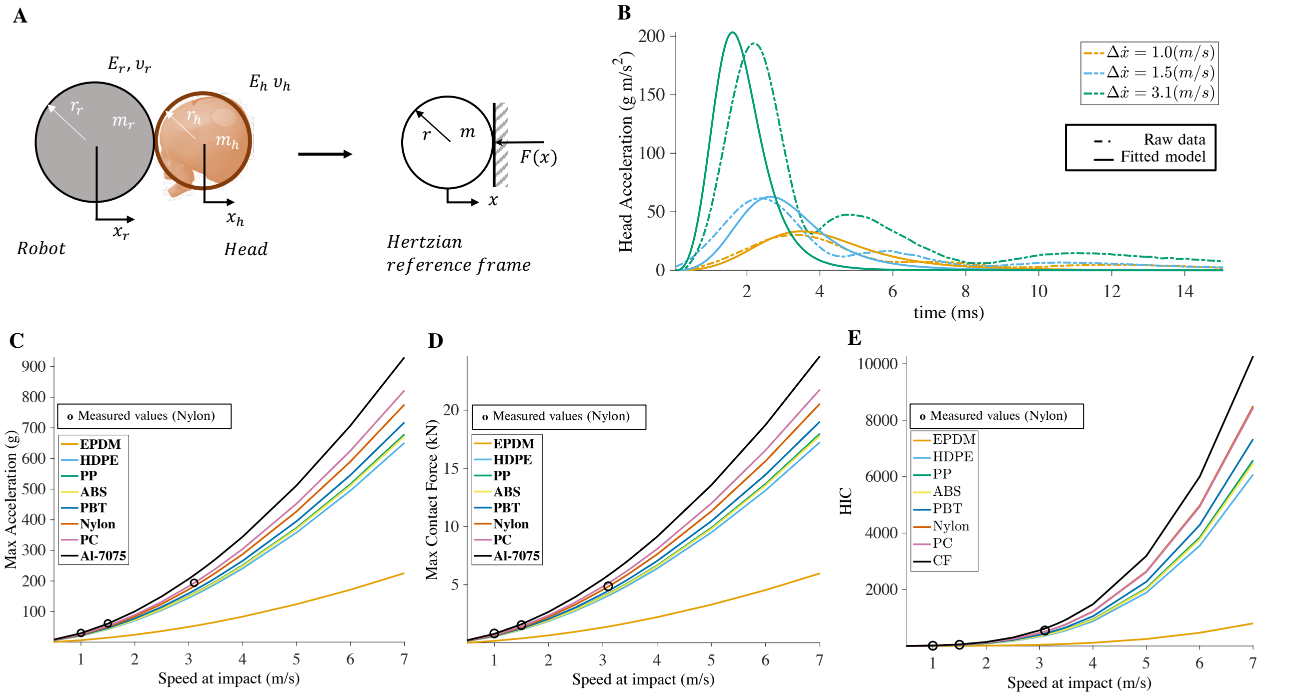

Supplementary Figure S1. **Collision model for head injury assessment.** (**A**) Hunt-Crossley contact model for head injury estimation[38,39]. (**B**) Comparison of raw head resultant acceleration data of the child-dummy collisions with the fitted model. (**C**) Results of simulated head acceleration when modifying the robot's hull material with common vehicle's bumper materials. (**D**) Corresponding impact forces when varying the robot's hull material. (**E**) Resultant HIC index when varying the robot's hull material.

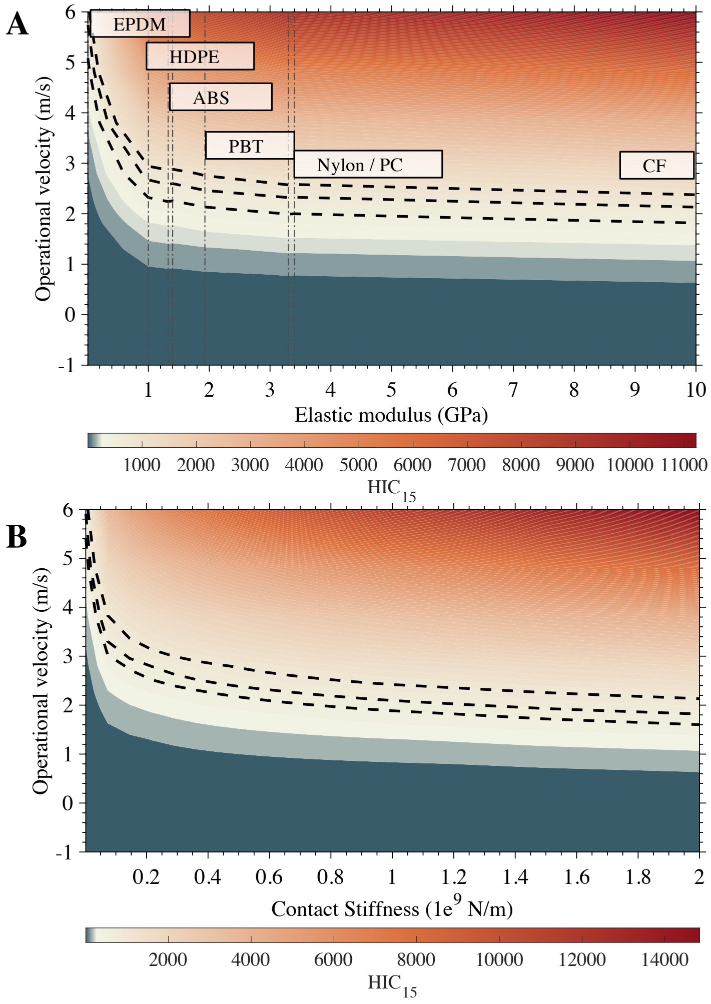

Supplementary Figure S2. Simulation results when varying robot's design parameters. (**A**) Impact severity (HIC index) as a function of the robot's surface material elastic modulus. (**B**) Effective surface contact stiffness effect on HIC index, presenting the limit velocities to mitigate HIC to under 5% p(AIS3+).

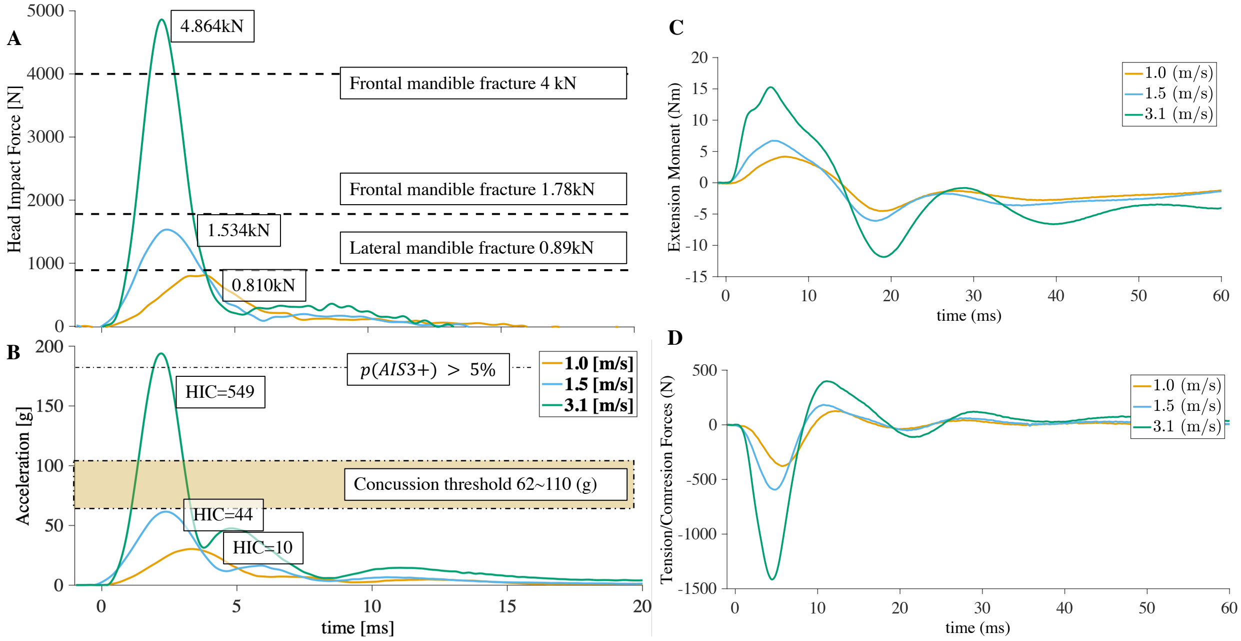

Supplementary Figure S3. Time-series data of child-dummy head injury. (**A**) Head impact force for the three measured impact speeds with reference to multiple bones measured fracture forces[40]. (**B**) Head acceleration profiles for each condition comparing known peak accelerations related to concussions[33].(**C**) Neck extension moments.(**D**) Neck tension/compression forces.

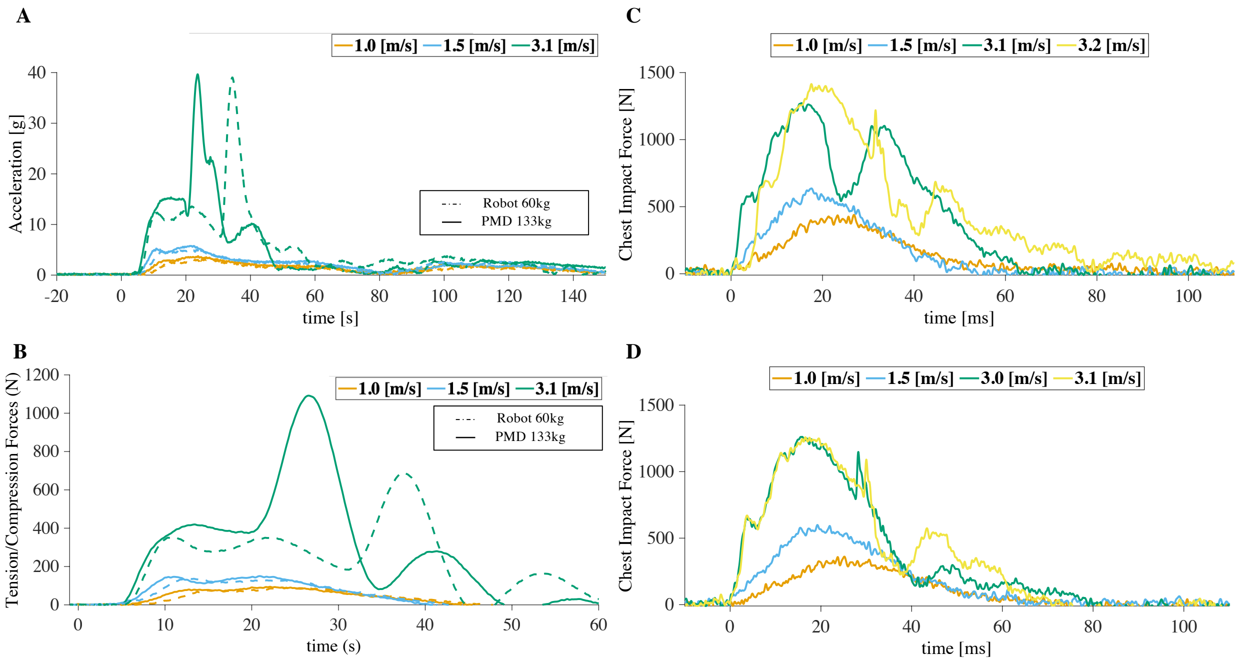

Supplementary Figure S4. **Times series data of child chest impacts:** (**A**) Thorax resultant acceleration for both tests with the robot at 133kg and 60 kg.

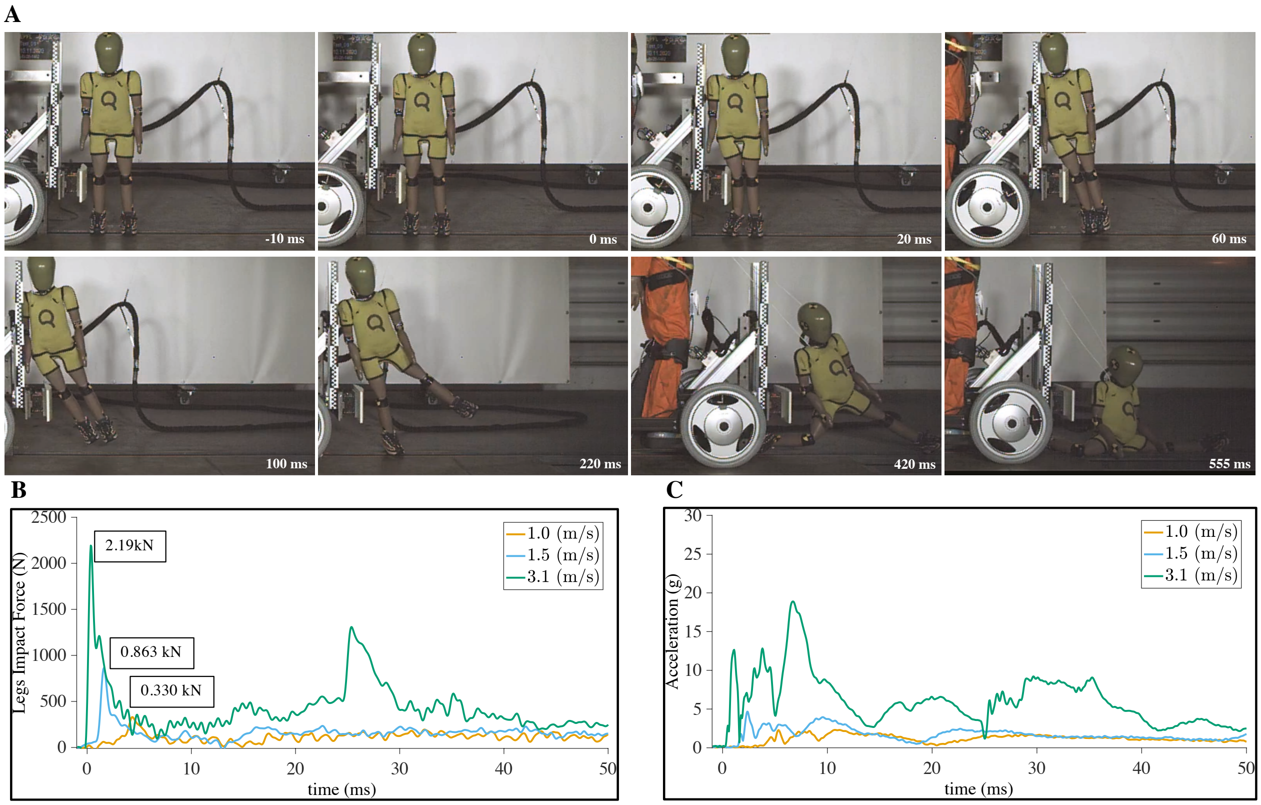

Supplementary Figure S5. Child dummy impact to the legs. (**A**) Time lapse of the collision at 3.1 m/s with the PMD. (**B**) Observed forces at the robot's bumper. (**C**) Observed pelvis acceleration induced to the child dummy.

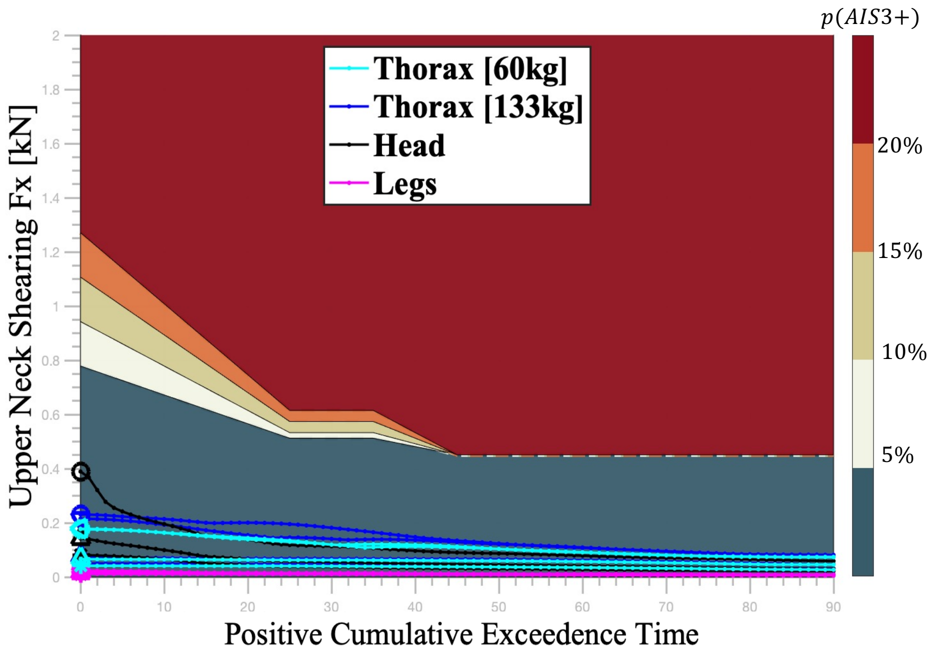

Supplementary Figure S6. **Neck injury analysis for shearing forces.** In this plot of the cumulative exceedance time of shearing neck forces (Fx), it is shown that for non of the frontal or lateral impacts no significant risk was found in this metric.

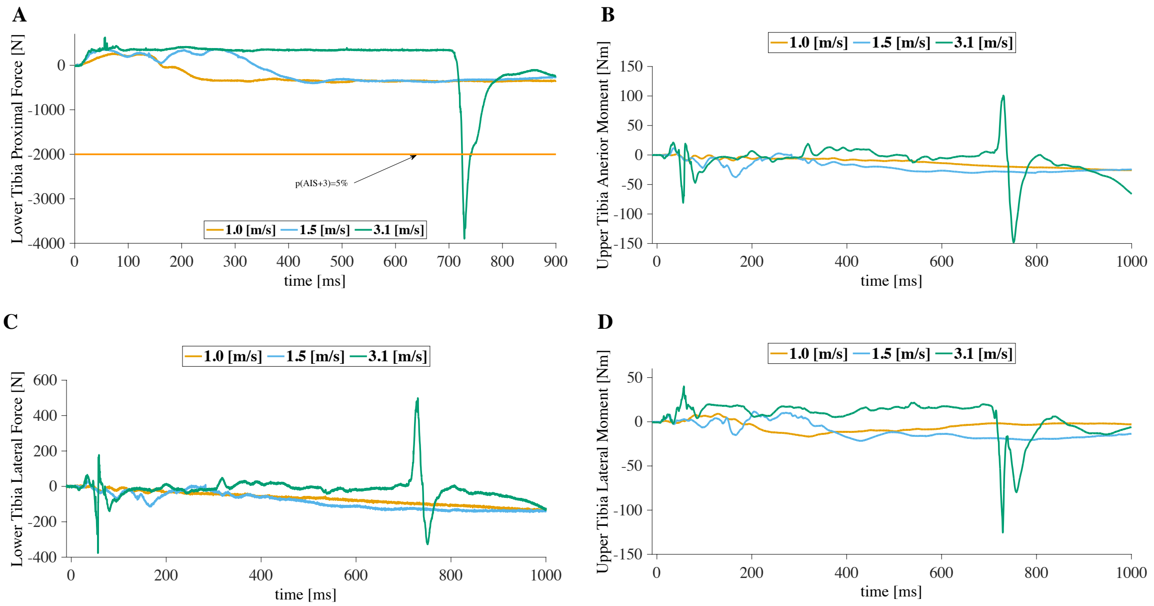

Supplementary Figure S7. Supplementary results for adult dummy indirectly impacted leg - left. (**A**) Lower tibia proximal force. (**B**) Upper tibia anterior moment. (**C**) Lower tibia lateral forces. (**D**) Upper tibia lateral moment.

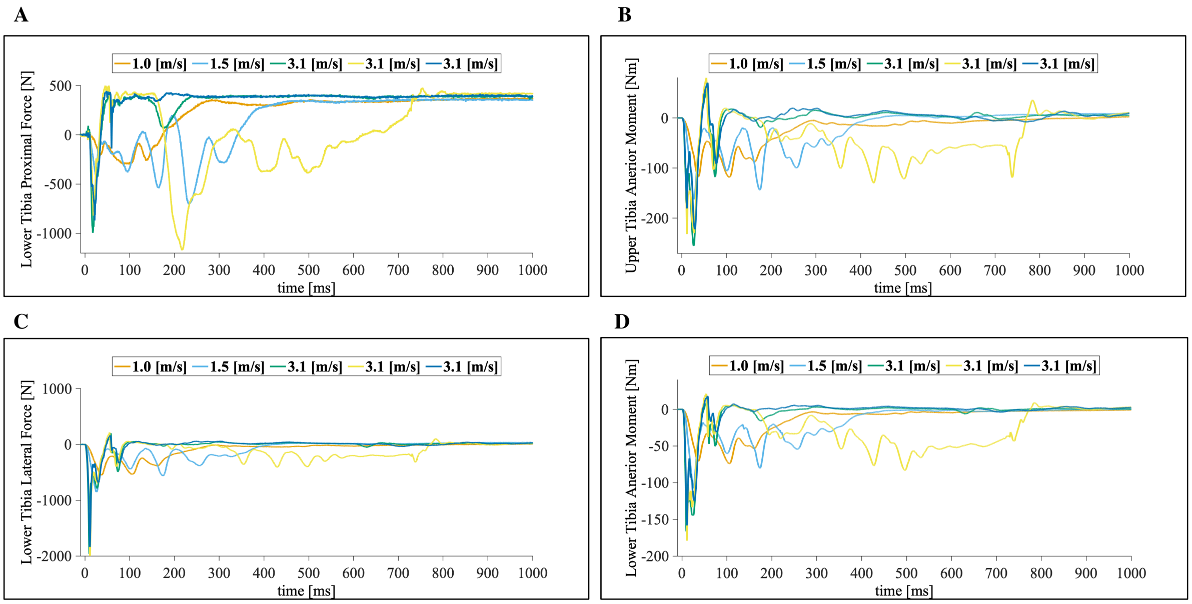

Supplementary Figure S8. Supplementary results for adult dummy directly impacted leg - right. (**A**) Lower tibia proximal force. (**B**) Upper tibia anterior moment. (**C**) Lower tibia lateral forces. (**D**) Upper tibia lateral moment.

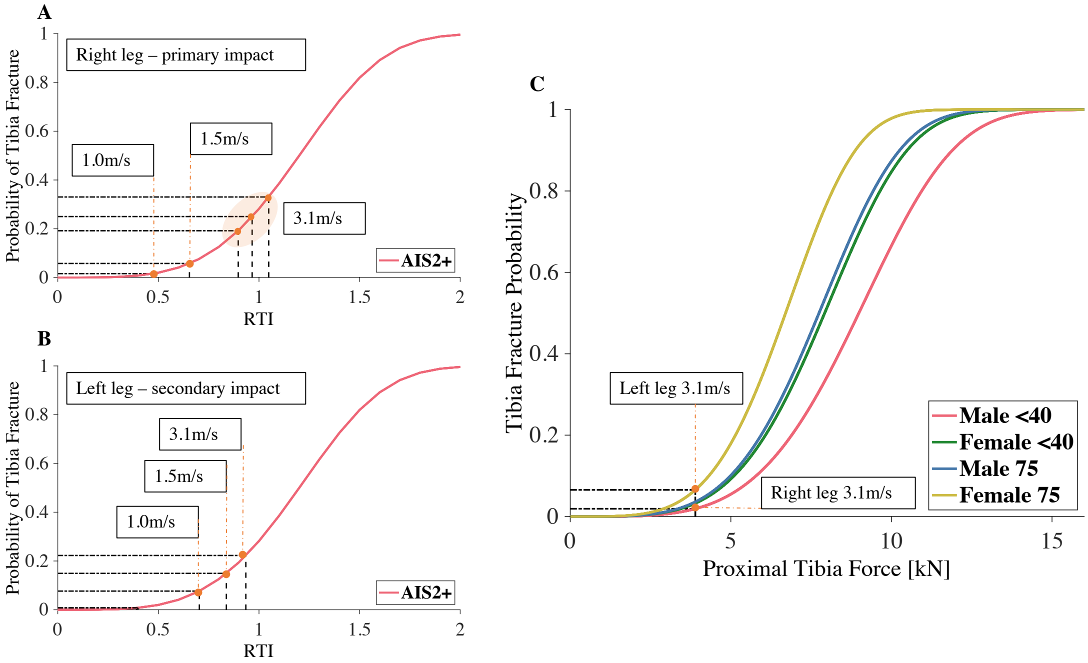

Supplementary Figure S9. Adult tibia injury probability. (**A**) Revised tibia index (RTI) data for the tested conditions over the probability of moderate injury (AIS2+) for the first impacted leg[11].(**B**) Same impacts' data over the injury probability for the indirectly impacted leg. (**C**) Estimated tibia fracture probably for different populations from the internal proximal tibia forces[12].

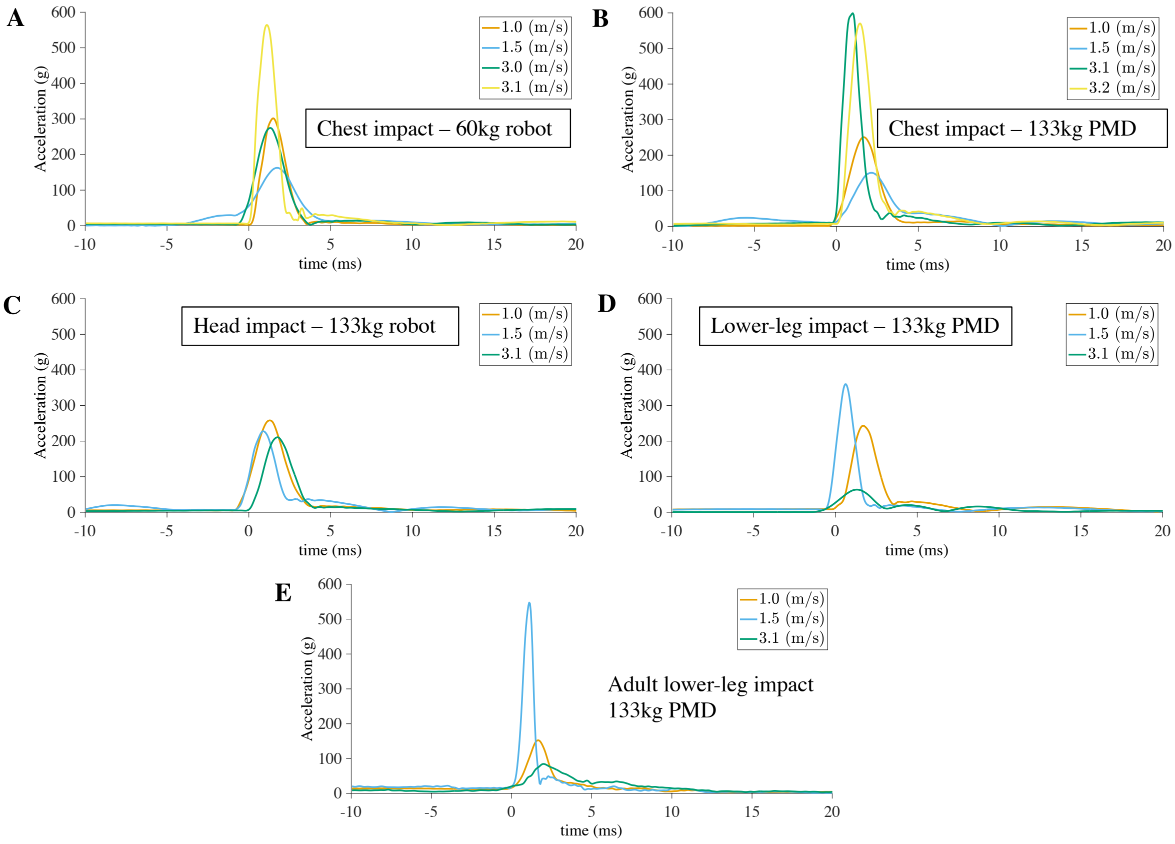

Supplementary Figure S10. Secondary head ground impacts. (**A**) MSR chest child impacts - 60kg. (**B**) PMD child chest impacts - 133kg. (**C**) PMD head impacts - 133kg. (**D**) PMD child leg impacts - 133kg. (**E**) PMD adult leg impacts - 133kg.

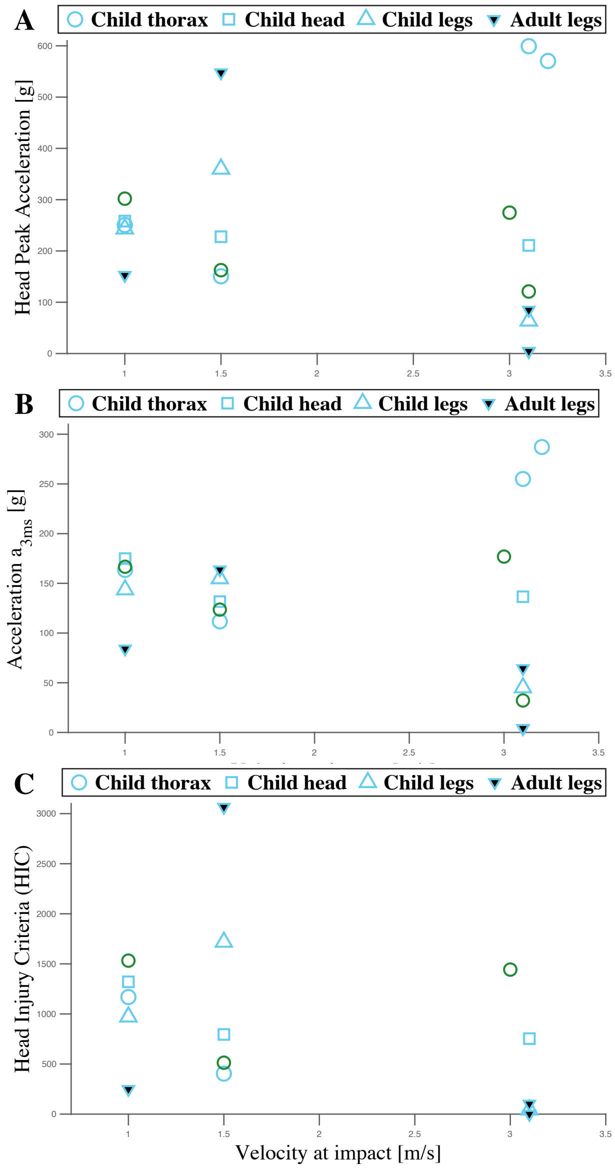

Supplementary Figure S11. Supplementary results of ground impacts. (**A**) Head peak acceleration for each impact condition for both adult and children.(**B**) Max cumulative 3ms acceleration for each impact condition. (**C**) Corresponding head injury criteria for each impact scenario.

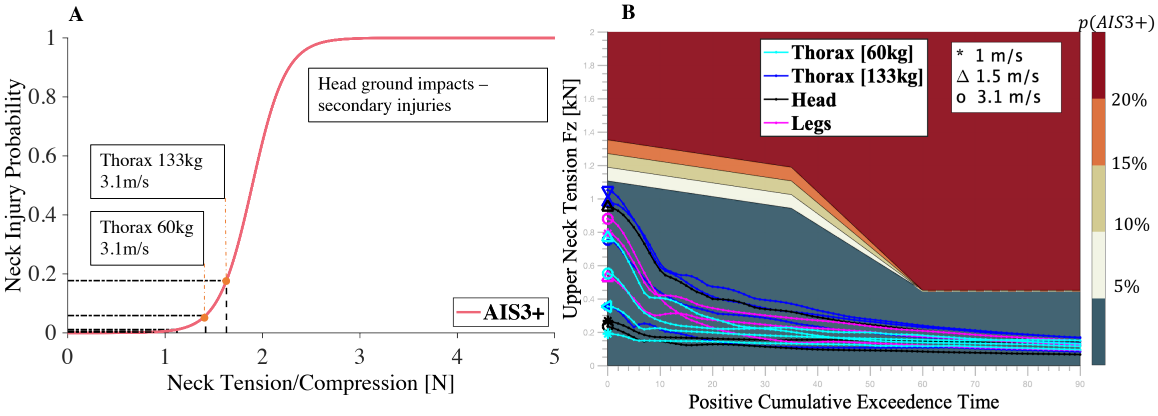

Supplementary Figure S12. **Supplementary results of ground impacts.** (**A**) Neck injuries observed during ground impacts, showing only relevant data over the 5% threshold of serious injury. (**B**) Cumulative neck tension forces, showing no significant probabilities of injury with values under 1.1kN .

Supplementary Video Captions:

Mobile service robots and personal mobility devices can now operate autonomously around pedestrians, at speed of 3.7 up to 18 mph, with masses ranging from 20 kg to 300kg. However, these devices are not standardised, and regulations differ per country and even within single countries.

We investigated the influence of operational speed and mass of a robot during impact with pedestrians, and contrasted risks faced by two different categories: children and adults.

We measured injuries to the head on blunt impacts at three speeds with an 133kg mobility device: At 3.1 m/s - accounting for the differential speed between a child running at 1.5 m/s colliding against a robot at 1.6 m/s. Head injury indicators showed a 14 % probability of a serious injury. In contrast, at the reduced impact speeds of 1.5 m/s and 1.0 m/s, all injury metrics were below the 5% risk threshold.

Chest impacts with the child dummy were recorded at the same 3 speeds of 3.1, 1.5 and 1.0 m/s.

As a highly instrumented body-part on the child dummy, we recorded the same impacts with a lighter robot of 60kg, representing smaller delivery robots.

We found a significant risk of serious injury with the chest deflection criteria, reaching up to 50 % probability of a serious injury for the 133kg robot impact compared to 32 % for the 60kg robot. At lower speeds of 1.5 and 1.0 m/s, no significant injuries were found.

The final tests were performed at the lower legs of children and adults. Results showed that all impacts, regardless of the speed, displayed a tibia index exceeding a 5% probability of tibia fracture. In this injury index, we found an increased probability of tibia fracture proportional to the speed at contact: 7.7 %, 14.9% and 33.2 % at the speeds of 1.0, 1.5 and 3.1 m/s respectively.

Although child injuries to lower-legs is not yet well documented, our data displayed up to 2.19 kN of impact force at 3.1 m/s. This is a force level comparative to the adult dummy, where impact forces over 1.4 kN showed over 15% probability of lower leg injury.

We found post-impact secondary injuries due to a fall to pose a significant risk of severe injuries to head and neck. With levels of injury higher than blunt impacts alone.

Contrasting head injury severity for the adult and child pedestrians, only one out of three trials in which the adult fell would have led to a serious head injury whereas for the child falls, 7 out of 14 lead to a serious injury.

Our results show a need to regulate robots and mobility devices through operational speed or by limit their usage in densely populated areas. This set of data and their analysis may also guide the design of robot controllers, and its navigation decision-making systems for mitigate risks of impact.

This opens a path for physically safe operations of autonomous robots in public environments through standards, traffic management, and legislation.

References

1. Li, X. & Kleiven, S. Improved safety standards are needed to better protect younger children at playgrounds. *Nat. Sci. Reports* **8**, 1–14, DOI: 10.1038/s41598-018-33393-z (2018).

2. Wismans, J. *et al.* Q-dummies Report: Advanced Child Dummies and Injury Criteria for Frontal Impact. Tech. Rep. 514, EEVC (2008).

3. Li, Z. *et al.* A statistical skull geometry model for children 0-3 years old. *PLoS ONE* **10**, 0–13, DOI: 10.1371/journal.pone.0127322 (2015).

4. NHTSA. FMVSS 213 Test Procedure - Child Restraint Systems. Tech. Rep. TP-213-10, DEPARTMENT OF TRANSPORTATION NATIONAL HIGHWAY TRAFFIC SAFETY ADMINISTRATION, Washington, DC. USA (2014).

5. Mertz, H. J., Prasad, P. & Irwin, A. L. Injury risk curves for children and adults in frontal and rear collisions. *SAE Tech. Pap.* DOI: 10.4271/973318 (1997).

6. Mertz, H. J., Irwin, A. L. & Prasad, P. Biomechanical and Scaling Basis for Frontal and Side Impact Injury Assessment Reference Values. *SAE Tech. Pap.* **47**, 155–188, DOI: 10.4271/2016-22-0018 (2003).

7. EuroNCAP. European New Car Assessment Programme (Euro NCAP) Assessment Protocol – Adult occupant protection. Tech. Rep. June, European New Car Assessment Programme (2020).

8. National Highway Traffic Safety Administration (NHTSA). New Car Assessment Program (NCAP). Tech. Rep., National Highway Traffic Safety Administration, USA (2008).

9. Palisson, A., Cassan, F., Trosseille, X., Lesire, P. & Alonzo, F. Estimating Q3 dummy injury criteria for frontal impacts using the child project results and scaling reference values. In *International IRCOBI Conference on the Biomechanics of Injury*, September, 263–276 (Netherlands, 2007).

10. Euro NCAP. Assessment protocol - Adult Occupant Protection v 7.0.3. Tech. Rep. November, European New Car Assessment Programme (2015).

11. Kuppa, S., Wang, J., Haffner, M. & Eppinger Rolf. Lower Extremity Injuries and Associated Injury Criteria. In *Proceedings of the Seventeenth International Technical Conference on the Enhanced Safety of Vehicles*, vol. 53, 1689–1699 (Amsterdam, 2001). arXiv:1011.1669v3.

12. Mildon, P. J., White, D., Sedman, A. J., Dorn, M. & Masouros, S. D. Injury Risk of the Human Leg Under High Rate Axial Loading. *Hum. Factors Mech. Eng. for Def. Saf.* **2**, DOI: 10.1007/s41314-018-0009-x (2018).

13. Prasad, P., Mertz, H. J., Dalmotas, D. J., Augenstein, J. S. & Digges, K. Evaluation of the Field Relevance of Several Injury Risk Functions. *SAE Tech. Pap.* **2010-Novem**, DOI: 10.4271/2010-22-0004 (2010).

14. Humanetics Innovative Solutions. Q3 (Advanced 3 year old child) User Manual User Manual. Tech. Rep. January, Humanetics Innovative Solutions Inc., Plymouth, USA (2016).

15. Rafukka, I. A., Sahari, B. B., Nuraini, A. A. & Manohar, A. A Modified Hybrid III 6-Year-Old Dummy Head Model for Lateral Impact Assessment. *Int. J. Veh. Technol.* **2016**, 1768512, DOI: 10.1155/2016/1768512 (2016).

16. Park, J. J., Haddadin, S., Song, J. B. & Albu-Schäffer, A. Designing optimally safe robot surface properties for minimizing the stress characteristics of Human-Robot collisions. In *Proceedings - IEEE International Conference on Robotics and Automation*, 5413–5420, DOI: 10.1109/ICRA.2011.5980282 (2011).

17. Unagi Inc. The Comprehensive Guide to Electric Scooter Laws (2021).

18. Japanese Industrial Standards Association. JIS T 9203 Electric Wheelchairs. Tech. Rep., Japanese Industrial Standards Association, Tokyo (2006).

19. of Transport Great Minister House, D. Mobility scooters and powered wheelchairs on the road (2021).

20. Council Regulation (EU). Regulation (EU) No 168/2013 of the European Parliament and of the Council of 15 quadricycles, 2013 on the approval and market surveillance of two- or three-wheel vehicles and quadricycles. *Off. J. Eur. Union* **60**, 52–128 (2013).

21. of Transport Great Minister House, D. Mobility scooters and powered wheelchairs on the road (2021).

22. and Tourism, M. o. L. I. T. Survey Results on the Use of Electric Wheelchairs Overseas Document 3. Tech. Rep., MLIT, Tokyo, Japan (2013).

23. Queensland Government. Rules for personal mobility devices (2019).

24. Willis, A., Gjersoe, N., Havard, C., Kerridge, J. & Kukla, R. Human movement behaviour in urban spaces: Implications for the design and modelling of effective pedestrian environments. *Environ. Plan. B: Plan. Des.* **31**, 805–828, DOI: 10.1068/b3060 (2004).

25. Carcreff, L. *et al.* Comparison of gait characteristics between clinical and daily life settings in children with cerebral palsy. *Sci. Reports* **10**, 1–11, DOI: 10.1038/s41598-020-59002-6 (2020).

26. Starship Inc. Starship delivery robot (2021).

27. ZMP Inc. Walking Speed Mobility - Rakuro ZMP (2021).

28. Aethon Inc. ST Engineering Aethon (2021).

29. Segway Inc. Segway Products (2021).

30. JD.com. E-commerce JD.com (2021).

31. Bluebotics Inc. Engeneering Services Bluebotics (2021).

32. Whill Inc. Whill Products (2021).

33. Campolettano, E. T. *et al.* Development of a Concussion Risk Function for a Youth Population Using Head Linear and Rotational Acceleration. *Annals Biomed. Eng.* **48**, 92–103, DOI: 10.1007/s10439-019-02382-2 (2020).

34. EuroNCAP. European New Car Assessment Programme (Euro NCAP) Assessment Protocol – Child occupant protection V.7.3.1. Tech. Rep. June, European New Car Assessment Programme (2020).

35. LinuxGizmos.com. Starship robot on the move (2021).

36. ZMP Inc. Delivery robot - Deliro ZMP (2021).

37. 1 Day Travel. Segway tour Utrecht (2021).

38. Hunt, K. & Crossley, E. Coefficient of restitution interpreted as damping in vibroimpact To cite this version : HAL Id : hal-01333795 Coefficient of Restitution Interpreted as Damping in Vibroimpact. *J. Appl. Mech. Am. Soc. Mech. Eng.* **42**, 440–445 (1975).

39. Haddadin, S., Albu-SchäCurrency Signffer, A. & Hirzinger, G. Requirements for safe robots: Measurements, analysis and new insights. *Int. J. Robotics Res.* **28**, 1507–1527, DOI: 10.1177/0278364909343970 (2009).

40. Hampson, D. Facial injury: A review of biomechanical studies and test procedures for facial injury assessment. *J. Biomech.* **28**, 1–7, DOI: 10.1016/0021-9290(95)80001-8 (1995).
